# Cardiac energy metabolic disorder and gut microbiota imbalance: a study on the therapeutic potential of Shenfu Injection in rats with heart failure

**DOI:** 10.3389/fmicb.2025.1509548

**Published:** 2025-02-25

**Authors:** Zhenyu Zhao, Zhixi Hu, Lin Li

**Affiliations:** ^1^Provincial Key Laboratory of TCM Diagnostics, Hunan University of Chinese Medicine, Changsha, China; ^2^Institute of TCM Diagnosis, Hunan University of Chinese Medicine, Changsha, China

**Keywords:** heart failure, gut microbiota, Shenfu Injection, short-chain fatty acids, myocardial energy metabolism, intestinal barrier function

## Abstract

**Objective:**

To investigate the relationship between heart failure (HF) and gut microbiota-mediated energy metabolism, and to explore the role of Shenfu Injection in this process.

**Materials and methods:**

In this study, Adriamycin-induced chronic heart failure (CHF) rat model was used and randomly divided into the blank control group (Normal, *n* = 9), HF control group (Model, *n* = 12), Shenfu Injection treatment group (SFI, *n* = 9), and positive drug control group (TMZ, *n* = 9). The changes in gut microbiota structure were analyzed by 16S rRNA high-throughput sequencing, the content of short-chain fatty acids (SCFAs) was detected by targeted metabolomics technology, and cardiac function and energy metabolism-related indicators were evaluated.

**Results:**

Myocardial energy metabolism in HF rats was disordered, characterized by reduced fatty acid oxidation, enhanced anaerobic glycolysis of glucose, mitochondrial damage, and decreased ATP content; The gut microbiota of HF rats was imbalanced, with a reduction in beneficial bacteria, an increase in conditional pathogenic bacteria, and impaired intestinal barrier function; Both Shenfu Injection and trimetazidine improved myocardial energy metabolism and cardiac function, but Shenfu Injection was more significant in regulating gut microbiota and improving intestinal health; The production of SCFAs from the gut microbiota of HF rats increased, which may be closely related to myocardial energy metabolism; SCFAs-producing bacteria Akkermansia and Blautia played a key role in the development of HF, and their abundance was positively correlated with SCFAs content.

**Conclusion:**

Shenfu Injection in treating HF may improve myocardial energy metabolism and intestinal health by regulating gut microbiota, especially the abundance of SCFAs-producing bacteria Akkermansia and Blautia, thereby exerting therapeutic effects. This provides theoretical support for treatment strategies based on gut microbiota.

## Introduction

Heart failure (HF), a clinical syndrome resulting from cardiac dysfunction, is the end stage of all cardiovascular diseases (CVDs), characterized by a reduction in the ability of the heart to pump and/or fill with blood, which cannot meet the metabolic needs of the body, resulting in symptoms and signs such as dyspnea, dizziness, fatigue, and fluid retention (Smith, 2017; Savarese et al., 2023). Globally, HF remains a significant clinical and public health issue (Youn et al., 2023). There are approximately 60 million HF patients worldwide, and HF is the leading cause of hospitalization for people over 65 (Becher et al., 2022; Engelfriet et al., 2011). And due to population growth and aging, the number of people with HF is expected to continue rising, bringing serious medical and socio-economic challenges (Groenewegen et al., 2020). With the in-depth understanding of the pathophysiological mechanism of HF, researchers began to focus on myocardial energy metabolism as a new target for treatment. The heart is a high-energy-consuming organ that requires a constant supply of energy to maintain its normal function. Mitochondria, known as the “energy factory” in cells, primarily produce energy through the process of oxidative phosphorylation, utilizing oxygen and nutrients such as glucose, fatty acids, and amino acids. The structural and functional integrity of mitochondria is crucial for the stability and adaptability of intracellular energy metabolism (Ni et al., 2015). Researchers have found that patients with HF generally have serious energy metabolism disorders, which are usually closely related to abnormal mitochondrial function, and then affect the systolic and diastolic functions of the heart, resulting in abnormal cardiac pump function and systemic energy metabolism failure (He et al., 2021).

There are about 3.8 × 10^13^ bacteria in the healthy human body, which can be found in the skin, oral cavity, nasopharyngeal cavity, intestine, urogenital tract, etc. (Sender et al., 2016). Most of them exist in the intestine (especially the colon), which is called “gut microbiota” (Guan and Sun, 2023). Under physiological conditions, the host and gut microbiota interact to form a complex mutualistic symbiotic relationship. The host offers an appropriate habitat along with essential nutrients to support the gut microbiota, while the flora aids the host in digesting food, synthesizing essential nutrients, and protecting against pathogenic bacteria invasion (Gao et al., 2023). When this homeostasis is disrupted, leading to an imbalance of the gut microbiota, it can be accompanied by a wide range of diseases, including inflammatory bowel disease (IBD) (Guo et al., 2022), diabetes (An et al., 2022), CVDs (Zou et al., 2022), and so on.

In recent years, the “intestinal hypothesis of HF” has been widely mentioned (Nagatomo and Tang, 2015), that is, in HF, the heart’s pumping function is abnormal, the cardiac output is reduced, and the blood circulation system is redistributed. The intestine, as the earliest organ of ischemia and hypoxia and the latest recovery, will appear ischemia–reperfusion injury. It can lead to intestinal wall ischemia/edema, intestinal epithelial barrier function destruction, intestinal permeability increase, and gut microbiota structure disorder, etc., which is conducive to the translocation of opportunistic pathogens and their metabolites into the bloodstream via the hepatic portal vein, thereby affecting the development of HF (Zhang et al., 2023; Luedde et al., 2017; Lupu et al., 2023; Tuerhongjiang et al., 2021). Li L. et al. (2021) observed the gut microbiota composition and metabolites of hypertensive HF rats induced by high salt diet and found that a total of 17 bacteria genera (e.g., Lactobacillus, Staphylococcus, Quinella, Peptococcus, etc.) and 35 metabolites (e.g., Uracil, Oleamide, Norvaline, Histamine, etc.) had significant changes. Sun et al. (2022) found that the abundance of firmicutes decreased significantly in patients with severe chronic HF (CHF), and Proteobacteria replaced Bacteroides to become the second most abundant microphyla in patients with severe CHF. In addition, the abundance of short-chain fatty acids (SCFAs) producing bacteria (such as Ruminococcaceae UCG-004, Ruminococcaceae UCG-002, etc.) decreased significantly. These findings suggest that gut microbiota is a potential therapeutic target for HF.

More and more studies have revealed the link between gut microbiota derivatives and HF, such as SCFAs (Zhao P. et al., 2022), trimethylamine-N-oxide (TMAO) (Tang et al., 2014), bile acids (BAs) (Eblimit et al., 2018), Urotoxins (Lekawanvijit et al., 2010), etc. Among them, SCFAs (mainly including acetic acid, propionic acid, butyric acid, etc.), as the main metabolites of gut microbiota, has received extensive attention in the regulation of energy homeostasis (Kimura et al., 2014; den Besten et al., 2013). Hu et al. (2020) found that acetate and butyrate can induce the transformation of mitochondria into the fusion process to improve mitochondrial damage, promote mitochondrial repair, reduce oxidative stress response, and improve mitochondrial energy metabolism in cells. Buchanan et al. (2023) found that changes in mitochondrial morphology (small and round, poorly differentiated cristae) and function (disruption of metabolic and homeostasis transcriptional regulation) of SH-SY5Y cells after propionic acid treatment were related to key mitochondrial regulatory factors such as cMYC, NRF1, TFAM, STOML2 and OPA1. Therefore, we can speculate that the gut microbiota may affect the function of mitochondria by controlling the production of SCFAs, and further interfere with the energy metabolism disorder of cardiomyocytes in the state of HF.

Currently, although Western medicine is the mainstream in the clinical practice of HF, traditional Chinese medicine (TCM) also plays an important role due to its remarkable efficacy in improving symptoms, enhancing exercise tolerance and improving quality of life (Chen et al., 2023; Yu Y. et al., 2019; Wang et al., 2022). In China, many TCM injections have been widely used in the clinical treatment of HF, and Shenfu Injection is one of them (Zheng et al., 2023; Wang et al., 2018). The formula, originating from Shenfu Decoction in the TCM classic “Ji Sheng Fang” of the Song Dynasty, is compounded using *Panax ginseng* C.A.Mey from the Araliaceae family, specifically the Ginseng radix et rhizoma rubra, and *Aconitum carmichaelii* Debx from the Ranunculaceae family, known as Aconiti lateralis radix praeparata. The primary active constituents are ginsenosides and aconitine (Xu et al., 2024). Modern pharmacological studies have revealed that it has anti-inflammatory, anti-oxidation, immunomodulatory, enhancing myocardial contractility and other cardiovascular-related effects (Ji et al., 2011; Zhang et al., 2016). Additionally, Yuan et al. (2015) discovered that in a porcine model, pre-administration of Shenfu Injection before ventricular fibrillation could ameliorate myocardial metabolism and reduce energy expenditure without exacerbating the excessive secretion of endogenous catecholamines. Yang et al. (2015) found that the characteristic changes of urine metabolomics in CHF rats were mainly manifested as disturbance of energy metabolism pathway, and SFD treatment could improve the cardiac function and reverse the metabolomic characteristics of CHF rats. In order to further explore the mechanism of action of Shenfu Injection, 16SrRNA high-throughput sequencing combined with targeted metabolomics technology was used in this study to clarify the potential relationship between the efficacy of Shenfu Injection in treating HF and the energy metabolism mediated by SCFAs producing bacteria.

## Materials and methods

### Animal

Fifty-four male Sprague–Dawley (SD) rats (6 weeks old, weighing 220 ± 10 g, SPF grade) were purchased from Hunan SJA Laboratory Animal Co., Ltd. The animal production license number is SCXK (Hunan) 2019–0004, and the animal quality certificate number is 430727221100675974. The rats were reared in SPF Laboratory of Laboratory Animal Center, Hunan University of Chinese Medicine. The study protocol was approved by the Experimental Animal Ethics Committee of Hunan University of Chinese Medicine (no. LLBH-202303140003). The design and implementation of the study followed the animal ethics guidelines formulated by the Ministry of Science and Technology of China. Before the experiment, all rats were fed adaptively for 1 week.

### Drugs, reagents, and instruments

Shenfu Injection [China Resources Sanjiu (Ya’an) Pharmaceutical Co., Ltd., no. 221111AK05], Trimetazidine Hydrochloride Sustained-release Tablets (Beijing Fuyuan Pharmaceutical, Zevex, no. H20065167), Doxorubicin (Shenzhen Wanle Pharmaceutical Co., Ltd., no. H44024359), Urethane (Shanghai Sourceleaf Biotechnology Co., Ltd., no. MFCD00007966), Acetic Acid (CCFD200033, CATO), Propionic Acid (CCFD200222, CATO), Isobutyric Acid (CCEM500589, CATO), Butyric Acid (CCFD200063, CATO), Isovaleric Acid (CCPD101342, CATO), Valeric Acid (CCFD200195, CATO), Isocaproic Acid (CCEM500417, CATO), Caproic Acid (CCFD200155, CATO), 2-Ethylbutyric Acid (B65222, CATO), Phosphoric Acid (10,015,418, Hushi), HPLC n-Butanol (A383-4, Fisher), FastPure Feces DNA Isolation Kit (Omega Bio-tek, Georgia, U.S.), FastPfu Polymerase (Beijing TransGen Biotech Co., Ltd.), PCR Machine (ABI GeneAmp®9,700, ABI, USA), Electrophoresis Apparatus (DYY-6C, Beijing Liuyi Biotechnology Co., Ltd.), VINNO 6 LAB Portable Digital Color Doppler Ultrasonic Diagnostic Instrument for Small Animals [FeiYino Technology (Suzhou) Co., Ltd.], GC/MSD Gas Chromatography–Mass Spectrometry Instrument (Agilent 8890B-5977B, Agilent), Multi-Sample Cryogenic Grinder (Shanghai Wanbai Biotechnology Co., Ltd., Wonbio-96c), Ultrasonic Constant Temperature Cleaning Machine (Ningbo Xinzhi Biotechnology Co., Ltd., SBL-10DT).

### Modeling and treatment

After adaptive feeding, the rats were divided into blank control group (Normal, *n* = 9) and model preparation group (*n* = 45) using a random number table. Rats in the model preparation group were intraperitoneally injected with adriamycin solution (concentration 2 mg/mL, volume 1.5 mL/kg) once a week for 7 weeks, while rats in the Normal group were intraperitoneally injected with the same amount of saline. According to the literature, the CHF model was established with an accumulated dose of doxorubicin at 21 mg/kg (Zhao Z. et al., 2022). After 7 weeks, the success of the model was judged according to the general behavioral signs and echocardiographic indicators.

**Figure 1:**
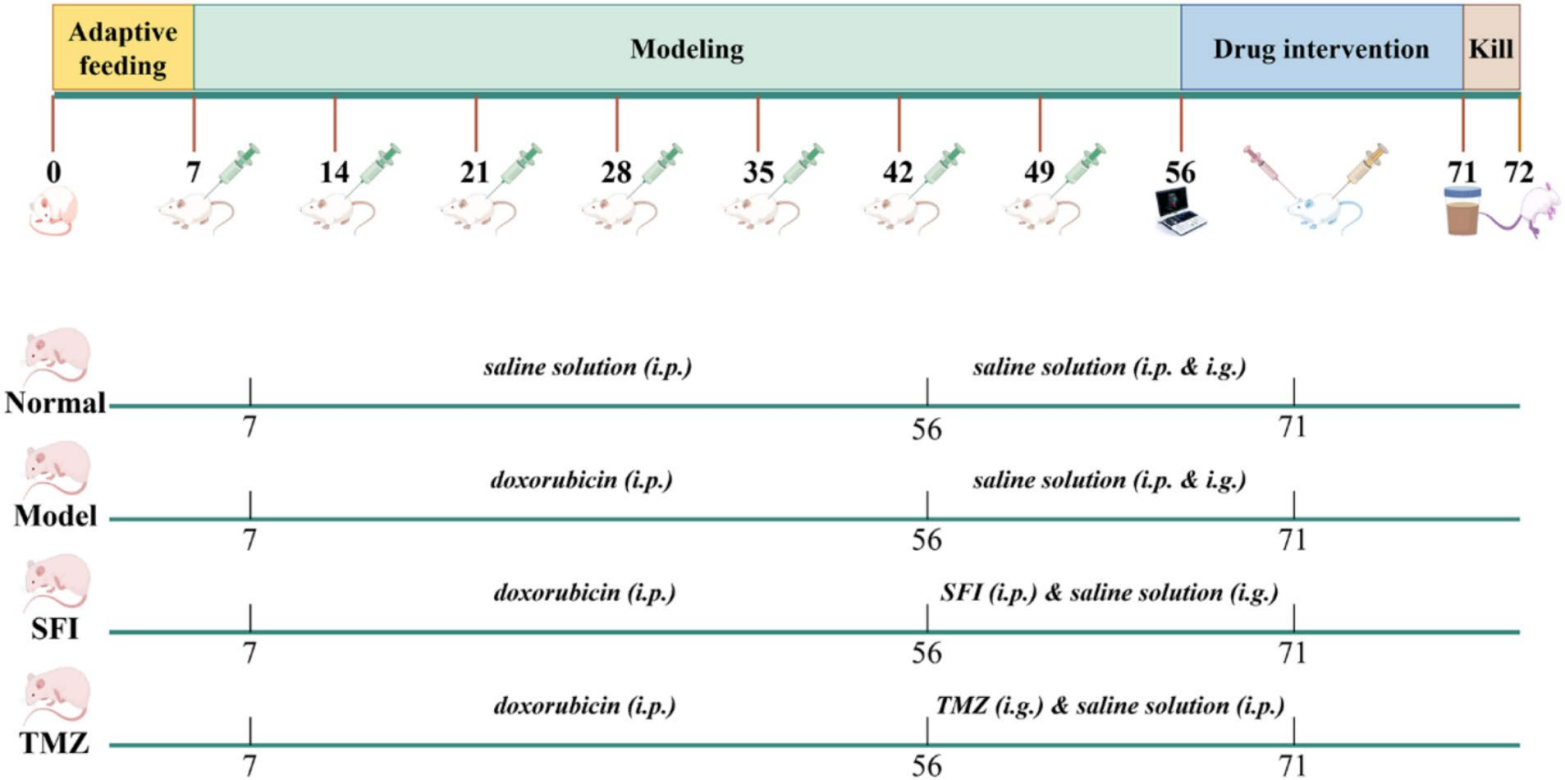
Experimental flow chart (this figure was drawn using Figdraw 2.0).

Excluding the dead rats and the unmodeled rats, a total of 30 rats were successfully modeled, and the success rate of the model was 66.67%. Subsequently, the rats with successful modeling were randomly divided into HF control group (Model, *n* = 12), Shenfu Injection treatment group (SFI, *n* = 9) and positive drug control group (TMZ, *n* = 9). Since TCM injections are not suitable for long-term use, rats in the four groups were treated for 15 days according to the previous administration method, as shown in Table 1 and Figure 1.

**Table 1:** Treatment protocols for rats in each group.

Group	Shenfu injection (i.p.)	Trimetazidine (i.g.)	Saline (i.p.)	Saline (i.g.)
Normal	*	*	6 mL/kg	5 mL/kg
Model	*	*	6 mL/kg	5 mL/kg
SFI	6 mL/kg	*	*	5 mL/kg
TMZ	*	5 mL/kg	6 mL/kg	*

Trimetazidine hydrochloride sustained-release tablet is a drug to optimize the energy metabolism of cardiomyocytes (Pușcaș et al., 2024), and is dissolved in saline to prepare a solution with a concentration of 2 mg/mL. * Indicates that the treatment would not be given.

### Sample collection

The day before tissue collection, fecal samples from each group of rats were collected into 2 mL cryovials, quick-frozen in liquid nitrogen, and then stored at −80°C for 16S rRNA gene sequencing and targeted metabolomics analysis. After the end of the drug intervention, all rats were anesthetized with urethane (concentration 0.2 g/mL, volume 5 mL/kg) in the morning of the next day, and echocardiography was performed first. Subsequently, blood samples were collected via the abdominal aorta, allowed to stand at room temperature for 2 h, and then centrifuged at 4°C, 3000 rpm for 15 min. The supernatant was collected into 2 mL cryovials and stored at −80°C for biochemical analysis. The rat hearts and colons were promptly excised. The heart tissue was flushed with saline, and part of the whole heart was placed in 4% paraformaldehyde for fixation at room temperature, to be used for HE staining. A tissue sample (2 mm × 2 mm) was excised from the left ventricle and immersed in electron microscopy fixative for 2 h at room temperature, then transferred to a 4°C refrigerator for storage, to be used for transmission electron microscopy. The remaining heart tissue was quick-frozen in liquid nitrogen and stored at −80°C for biochemical analysis. The colonic tissue was first scraped for the mucosal layer using a coverslip, the chyle was extruded, washed with saline, and part of it was fixed in 4% paraformaldehyde at room temperature for HE staining. Another part was quick-frozen in liquid nitrogen and stored at −80°C for Western Blot analysis.

### Echocardiography

After anesthesia, the rats were secured to a mouse board with rubber bands, and the hair in the area of the rat’s heartbeats was removed using hair removal cream. Using rat ultrasound system, an appropriate amount of coupling agent was applied to the rat probe. Under two-dimensional ultrasound guidance, an M-mode linear array probe was used to obtain the long-axis view of the left ventricle. The left ventricular ejection fraction (LVEF) and left ventricular fractional shortening (LVFS) were calculated using the eight-point method to assess left ventricular systolic function.

### Hematoxylin–eosin staining

The heart and colon tissues fixed with 4% paraformaldehyde were dehydrated and defatted before being immersed in liquid paraffin. They were then cut into thin sections using a microtome and flattened onto glass slides. After defatting the wax slides, the slides were immersed in Hematoxylin staining solution and Eosin staining solution, respectively, and sealed after dehydration and transparency.

### Transmission electron microscopy examination of myocardial tissue

The fixed heart was rinsed with phosphate buffer solution (PBS) for 30 min, followed by fixation in a dark room with 1% osmium tetroxide for 2 h. It was then dehydrated sequentially with gradient ethanol solutions (30–50%-70–90–100%), infiltrated with epoxy resin, embedded, and cut into ultra-thin sections (60–80 nm). The sections were stained with uranyl acetate and lead citrate, and observed and photographed under a transmission electron microscope.

### Western blot analysis of colon tissue

Protein extraction: take 0.025 g of colon tissue, wash with ice-cold PBS, add 300 μL of RIPA lysis buffer, and homogenize in a homogenizer until no tissue chunks are visible; lyse on ice for 10 min, centrifuge at 4°C and 12,000 rpm for 15 min, and transfer the supernatant to a 1.5 mL tube. Western Blot: Prepare the gel (10% separation gel+4.8% stacking gel), sample preparation (200 μL of protein+50 μL of 5*loading buffer, boil for 5 min, and rapidly cool on ice), electrophoresis (75 V, 130 min), membrane transfer (cut the full membrane, 300 mA constant current), blocking (5% non-fat dry milk, room temperature for 90 min), primary antibody incubation (overnight at 4°C, 30 min at room temperature, see Supplementary Table 1 for dilution ratios), secondary antibody incubation (room temperature for 90 min, see Supplementary Table 1 for dilution ratios), color development/exposure (ECL chemiluminescent solution, imaging).

### Biochemical analysis

According to the instructions, the content of pyruvic acid (PA), lactic acid (LA), non-esterified fatty acid (NEFA), malondialdehyde (MDA), superoxide dismutase (SOD), and adenosine triphosphate (ATP) in rat heart tissue, as well as the expression level of N-terminal pro-brain natriuretic peptide (NT-proBNP) in rat serum, were detected using the corresponding kit.

### 16S rRNA gene sequencing

#### DNA extraction and PCR amplification

Genomic DNA from the microbial community in fecal samples was isolated using the FastPure Feces DNA Isolation Kit, following the protocol provided by the manufacturer. The extracted DNA was analyzed on a 1% agarose gel, and its concentration along with purity was assessed using a NanoDrop 2000 UV–vis spectrophotometer. In the context of bacterial communities, the 16S rRNA genes of bacteria were selectively amplified with the general bacterial primers 27F (5’-AGRGTTYGATYMTGGCTCAG-3′) and 1492R (5’-RGYTACCTTGTTACGACTT-3′) as reported in reference (Weisburg et al., 1991). To differentiate between samples, the primers were appended with PacBio barcode sequences. The amplification mixtures, with a total volume of 20 μL, comprised 4 μL of 5 × FastPfu buffer, 2 μL of 2.5 mM dNTPs, 0.8 μL of forward primer at 5 μM, 0.8 μL of reverse primer at 5 μM, 0.4 μL of FastPfu DNA Polymerase, 10 ng of template DNA, and an appropriate amount of DNase-free water to complete the volume. The PCR amplification process was conducted as outlined: starting with a preliminary denaturation step at 95°C for a duration of 3 min, this was succeeded by 29 cycles consisting of denaturation at 95°C for 30 s, annealing at 60°C for 30 s, and elongation at 72°C for 45 s. A final extension was carried out at 72°C for 10 min, concluding with a cool-down to 10°C. The PCR was replicated three times. Subsequent to electrophoresis, the amplified PCR products were cleansed using AMPure^®^ PB beads and their concentration was determined using the Quantus™ Fluorometer.

### DNA library construction and sequencing

The purification of products was followed by their combination in equal molar ratios to assemble the DNA library, utilizing the SMRTbell Prep Kit 3.0 as per PacBio’s guidelines. Subsequently, the purified SMRTbell libraries were subjected to sequencing on the Pacbio Sequel IIe System, a service provided by Majorbio Bio-Pharm Technology Co. Ltd. located in Shanghai, China.

### Processing of sequencing data

The raw sequencing data from PacBio was subjected to analysis with SMRTLink software (version 11.0) to yield high-fidelity (HiFi) reads that met the criteria of at least three complete passes and a sequence accuracy of 99%. These HiFi reads were then demultiplexed based on barcodes and filtered by length. In the case of the bacterial 16S rRNA gene sequences, those shorter than 1,000 base pairs or longer than 1,800 base pairs were excluded.

The HiFi reads were grouped into operational taxonomic units (OTUs) using UPARSE 11 (Edgar, 2013), with a sequence similarity threshold set at 97%. The representative sequence for each OTU was chosen based on abundance. The OTU table was then curated manually to exclude chloroplast sequences across all samples. To mitigate the impact of sequencing depth on alpha and beta diversity metrics, the count of 16S rRNA gene sequences per sample was standardized to a depth of 8,185. Taxonomic classification of the representative OTU sequences was conducted using the RDP Classifier version 2.13 (Wang et al., 2007), referencing the 16S rRNA gene database (Silva v138), with a confidence level cutoff of 0.7.

### Bioinformatics analysis

Rarefaction curves and alpha diversity indices were calculated with Mothur v1.30.2 (Schloss et al., 2009). Principal co-ordinates analysis (PCoA), non-metric multidimensional scaling (NMDS) analysis, Venn plot, Bar plot, Pie plot, neutral community model (NCM) analysis, Variance Inflation Factor (VIF) analysis, spearman correlation analysis, regression analysis, ROC analysis, Mantel-test network were completed by R language (version 3.3.1). The Circos diagram was drawn by Circos-0.67-7 software.^1^ GMHI index and MDI index were based on the literature (Gunathilake et al., 2020; Gupta et al., 2020) to calculate. Linear discriminant analysis (LDA) (Segata et al., 2011)^2^ was made by LEfSe software. Testing for between-group differences was performed with the use of R (version 3.3.1) and python. The co-occurrence networks were produced by Networkx software (version 1.11) (Barberán et al., 2012). In addition, we would like to express our gratitude to Shanghai Meiji Biomedical Technology Co., Ltd. for their technical support.

### Targeted metabolomics analysis

#### SCFAs standard solution preparation

Preparation of mixed standard: 9840 μL n-butanol (HPLC grade) was loaded into a 15 mL centrifuge tube, and an appropriate amount of 8 kinds of SCFAs standard products were added in turn, vortexed and mixed to obtain the mixed standard stock solution A of 8 kinds of SCFAs. Preparation of internal standard: 9990 μL of n-butanol (HPLC grade) was loaded into a 15 mL centrifuge tube, and 10 μL of internal standard 2-ethylbutyric acid was added, vortexed and mixed to obtain internal standard stock solution B. The above mixed standard solutions A and B were diluted into 8 different concentrations of working solutions with n-butanol, and then loaded into sample vials for GC–MS detection and analysis.

### Sample processing

Weigh 20 mg of fecal sample into a 2 mL grinding tube, add 800 μL of 0.5% phosphoric acid solution (containing 10 μg/mL of internal standard 2-ethylbutyric acid). Freeze-grind the sample for 3 min (50 Hz), then sonicate for 10 min. Centrifuge at 4°C and 13,000 g for 15 min. Transfer 200 μL of the supernatant to a 1.5 mL centrifuge tube, and then add 200 μL of n-butanol solvent for extraction. Vortex for 10 s, sonicate at low temperature for 10 min, centrifuge at 4°C and 13,000 g for 5 min. Transfer the supernatant solution to a sample vial for analysis.

### GC–MS detection

Chromatographic conditions: HP FFAP capillary column was used with high purity helium gas (purity not <99.999%) as the carrier gas at a flow rate of 1.0 mL/min. The injector temperature was set at 180°C. The sample size was 1 μL with split injection at a split ratio of 10:1, and the solvent delay was 2.5 min. Programmed heating: the initial column oven temperature was 80°C, ramped up to 120°C at 20°C/min, then to 160°C at 5°C/min, followed by holding at 220°C for 3 min. Mass spectrometry conditions: electron impact ion source (EI) was used with an ion source temperature of 230°C, a quadrupole temperature of 150°C, and a transfer line temperature of 230°C. The electron energy was 70 eV. The scanning mode was selected ion monitoring (SIM).

### Data analysis

The target SCFAs ion fragments were automatically identified and integrated using the default parameters of the Masshunter quantitation software (v10.0.707.0, Agilent Technologies, USA), with manual inspection as an auxiliary step. The detection concentrations of each sample were calculated through standard curves, and the actual content of SCFAs in the samples was converted accordingly. Multivariate statistical analyses were performed using the R language.

### Quality control of Shenfu Injection

The active components of Shenfu Injection were analyzed using non-targeted metabolomics technology. The metabolites in the samples were detected using an ultra-high performance liquid chromatography-tandem orbitrap mass spectrometer (UHPLC-Q Exactive HFX, Thermo, USA). Qualitative and quantitative analysis of metabolites in biological samples was performed by matching the retention time, molecular mass (molecular mass error within 10 ppm) and secondary fragmentation spectrum of metabolites in the self-established Chinese herbal medicine database.

### Sample preparation and extraction

The sample were thawed slowly at 4°C, and the right sample (0.5–1.0 mL) were taken into the centrifuge tube, added 2 times the volume of the extraction solution (methanol/acetonitrile, 1:1, v/v), homogenized 60 s, extracted 30 min by low-temperature ultrasonic, centrifuged 10 min with 12,000 rpm at 4°C, set still for 1 h to precipitate protein at −20°C, then centrifuged 10 min with 12,000 rpm at 4°C, the supernatant solution were dried in vacuum, added 0.1 mL 50% acetonitrile solution, homogenized and centrifuged 10 min with 12,000 rpm at 4°C, the supernatant were taken for computer detection.

### UPLC conditions

The specimens obtained were subjected to analysis with an UPLC-Orbitrap-MS setup (UPLC, Vanquish; MS, HFX). The parameters for analysis were as follows: UPLC specifications included a Waters HSS T3 column (100 × 2.1 mm, 1.8 μm); a column temperature of 40°C; a flow rate of 0.3 mL/min; a 2 μL injection volume; and a mobile phase consisting of Milli-Q water with 0.1% formic acid (phase A) and acetonitrile with 0.1% formic acid (phase B). The gradient program was as follows: starting at 0 min with phase A/phase B at 100:0 (v/v), holding for 1 min at 100:0 (v/v), then changing to 5:95 (v/v) by 12 min and maintaining until 13 min, returning to 100:0 (v/v) by 13.1 min, and ending at 17 min with phase A/phase B at 100:0 (v/v).

### LC–MS/MS analysis

Human Resource Management System (HRMS) data were documented using a Q Exactive HFX Hybrid Quadrupole Orbitrap mass spectrometer, fitted with a heated Electrospray Ionization (ESI) source from Thermo Fisher Scientific. The data acquisition was performed with the Full-ms-ddMS2 method. The parameters for the ESI source were adjusted as follows: sheath gas pressure at 40 arbitrary units, auxiliary gas pressure at 10 arbitrary units, spray voltage at +3,000 volts/ -2800 volts, source temperature at 350°C, and ion transport tube temperature at 320°C. The primary mass spectrometry scan range (m/z) extended from 70 to 1,050 Daltons, featuring a primary resolution of 70,000 and a secondary resolution of 17,500.

### Statistical analysis

GraphPad Prism software (version 8.0.2) was used for statistical analysis. For the univariate measurement data of two independent samples, if it was in accordance with normality and homogeneity of variance, unpaired t test was used. Unpaired t’ test was used if the results were normal but the variance was not homogeneous. If the data did not conform to normality and were the original observation data of measurement data, the Wilcoxon rank sum test was used. For multiple samples of univariate measurement data, if they were in accordance with normality and homogeneity of variance, one-way analysis of variance was used. If normality or homogeneity of variance was not met, the Kruskal-Wallis H test was used.

## Results

### Quality control of Shenfu injection

**Figure 2:**
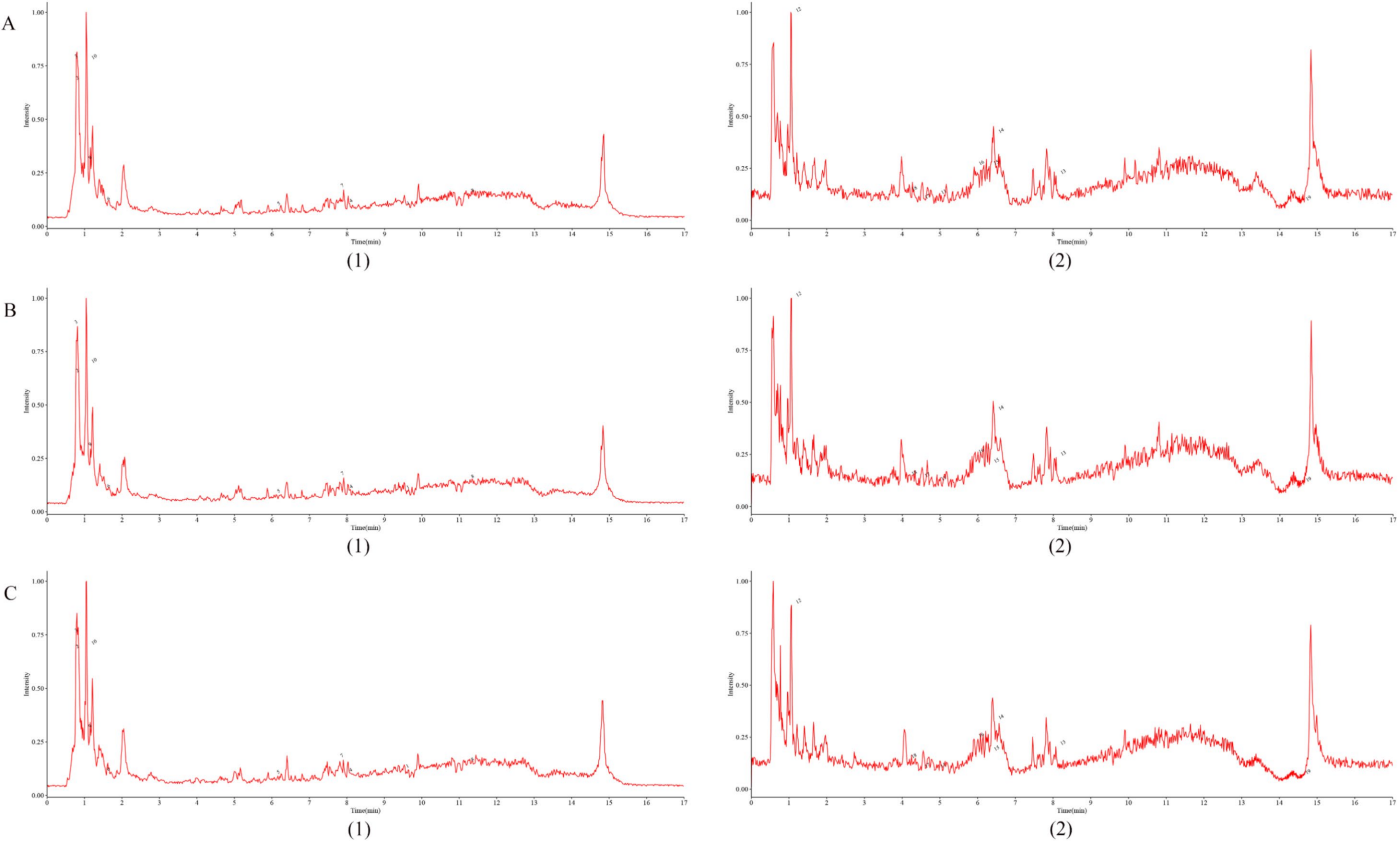
Fingerprint of Shenfu Injection. **(A–C)** represent the results of three random sampling tests, respectively, (1)-negative; (2)-positive.

Based on the results of non-targeted metabolomics detection (Supplementary Table 2), and by searching for the active ingredients of Shenfu Injection in the BATMAN-TCM database,^3^ the overlapping substances were marked in Figure 2 (Details are provided in the Supplementary Table 3).

### Morphological observation

The rats in the Normal group had good mental state, active behavior, neat and shiny fur, no vertical hair arched back, no cringe, and normal stool and urine. The rats in the Model group were listless and unresponsive, their fur was loose and dull, their bodies were curled up, with obvious clumping phenomenon, obvious ascites, and loose stool. In the SFI group, the spirit of rats became better, the movement was more sensitive, the fur was yellow and soft, the phenomenon of cringe and clumps was reduced, ascites was slightly relieved, and the symptoms of loose stool were relieved. In the TMZ group, the spirit of the rats was improved, the activity was increased, the hair was not significantly improved, the cringe and clumps were reduced, and the symptoms of ascites and diarrhea were improved.

### Cardiac structure and function

Echocardiography was used to evaluate the cardiac function of rats in each group. The results showed that compared to the Normal group, the Model group had significantly lower values of LVEF, LVFS, LVPWd, LVPWs, IVSd, and IVSs, as well as significantly higher values of LVIDd, LVIDs, LVEDV, and LVESV. These findings suggest that the left ventricular myocardial contractility of rats in the Model group was significantly weakened, with a decrease in cardiac output and insufficient tissue perfusion. Additionally, with LVFS <30%, it indicates that the HF model was successfully established, showing obvious functional changes of CHF.

**Figure 3:**
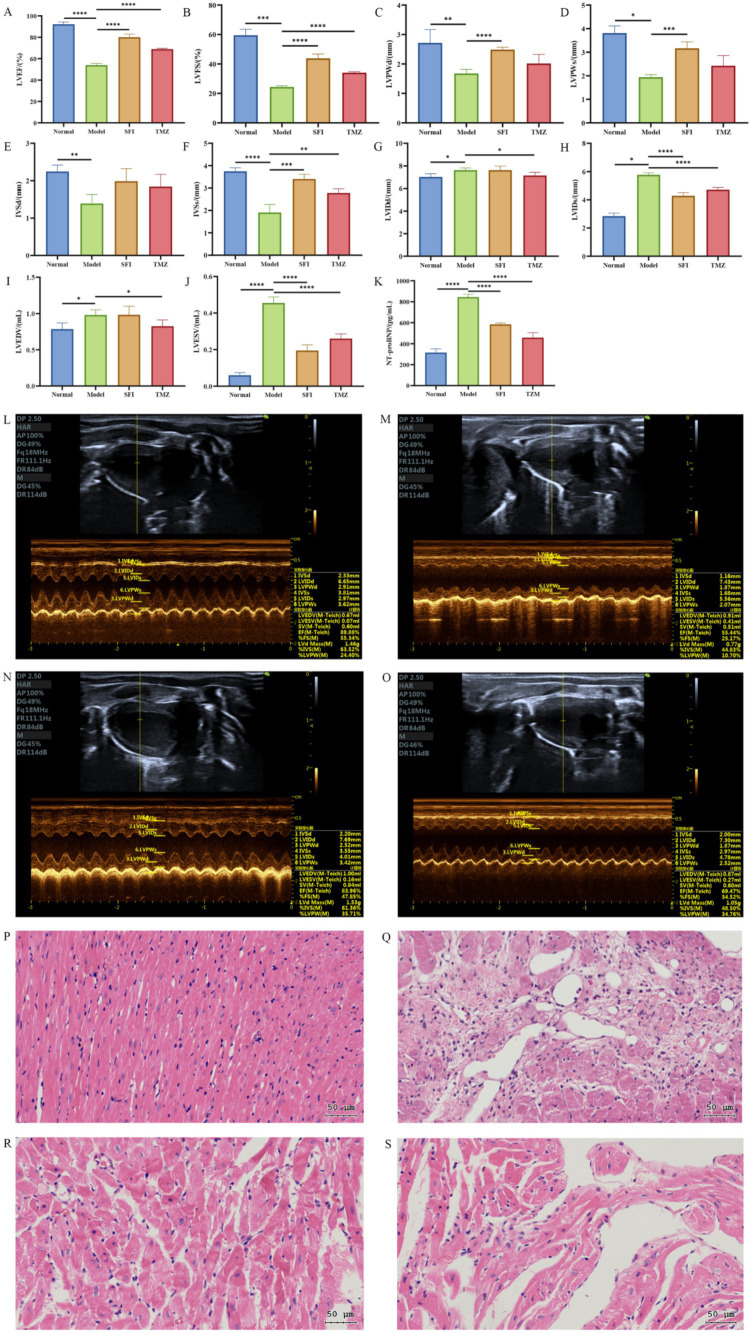
Cardiac indicators. **(A)** left ventricular ejection fraction, LVEF; **(B)** left ventricular fraction shortening, LVFS; **(C)** left ventricular posterior wall dimensions, LVPWd; **(D)** left ventricular posterior wall thickness in systole, LVPWs; **(E)** interventricular septum thickness at end-diastole, IVSd; **(F)** interventricular septum thickness at end-systole, IVSs; **(G)** left ventricular internal dimension in diastole, LVIDd; **(H)** left ventricular internal dimension in systole, LVIDs; **(I)** left ventricular end-diastolic volume, LVEDV; **(J)** left ventricular end-systolic volume, LVESV; **(K)** N-Terminal pro-Brain Natriuretic Peptide, NT-proBNP; **(L–O)** Echocardiography. **(K)** Normal group, **(L)** Model group, **(M)** SFI group, **(N)** TMZ group; **(P–S)** Myocardial tissue HE staining (The scale bar is 50 microns). **(P)** Normal group, **(Q)** Model group, **(R)** SFI group, **(S)** TMZ group. *p < 0.05; **p < 0.01; ***p < 0.001; ****p < 0.0001. Normal, Model, SFI and TMZ represent the blank control group, HF control group, Shenfu Injection treatment group and positive drug control group, respectively.

Compared to the Model group, the SFI group showed significant increases in LVEF, LVFS, LVPWd, LVPWs, and IVSs values, a trend of rebound in the IVSd value, and significant decreases in LVIDs and LVESV values, indicating that the Shenfu Injection has a good effect on improving the cardiac function of HF rats. Although the slight increase in LVIDd and LVEDV values appears to be a negative change, when assessed in conjunction with other indicators, the cardiac function of rats in the SFI group has improved. Compared to the Model group, the TMZ group showed significant increases in LVEF, LVFS, and IVSs values, a trend of rebound in LVPWd, LVPWs, and IVSd values, and significant decreases in LVIDd, LVIDs, LVEDV, and LVESV values, suggesting that trimetazidine has a therapeutic effect on HF rats (Figures 3A–J,L).

The myocardial fibers in the Normal group were arranged orderly, with an intact structure. The cytoplasm staining was uniform, and the nuclear contours were distinct. No pathological changes such as inflammatory cell infiltration, edema, or necrosis were observed. In the Model group, the myocardial tissue showed evident myofiber dissolution and inflammatory cell infiltration. The cytoplasm staining was uneven, and myocardial cell necrosis was visible. There were no obvious signs of inflammatory cell infiltration and myofibrolysis in the SFI group. Most of the nuclei were round or oval, located in the center of the cell, and the nuclear membrane was clear. The cytoplasm was more evenly distributed, with slightly light staining in some areas and enlarged nuclei, which were considered to have mild cellular edema. In the TMZ group, the intercellular substance in the myocardial tissue was reduced, with a small amount of collagen and elastic fibers distributed within. In some areas, inflammatory cell infiltration was visible, but no obvious necrotic areas were observed (Figures 3P–S).

The result of the blood indicator NT-proBNP also confirmed the success of the HF model (Figure 3K). Compared with the Normal group, the NT-proBNP levels in the Model group significantly increased, and after treatment, the NT-proBNP levels in the SFI group and TMZ group significantly decreased.

### Colonic structure and function

**Figure 4:**
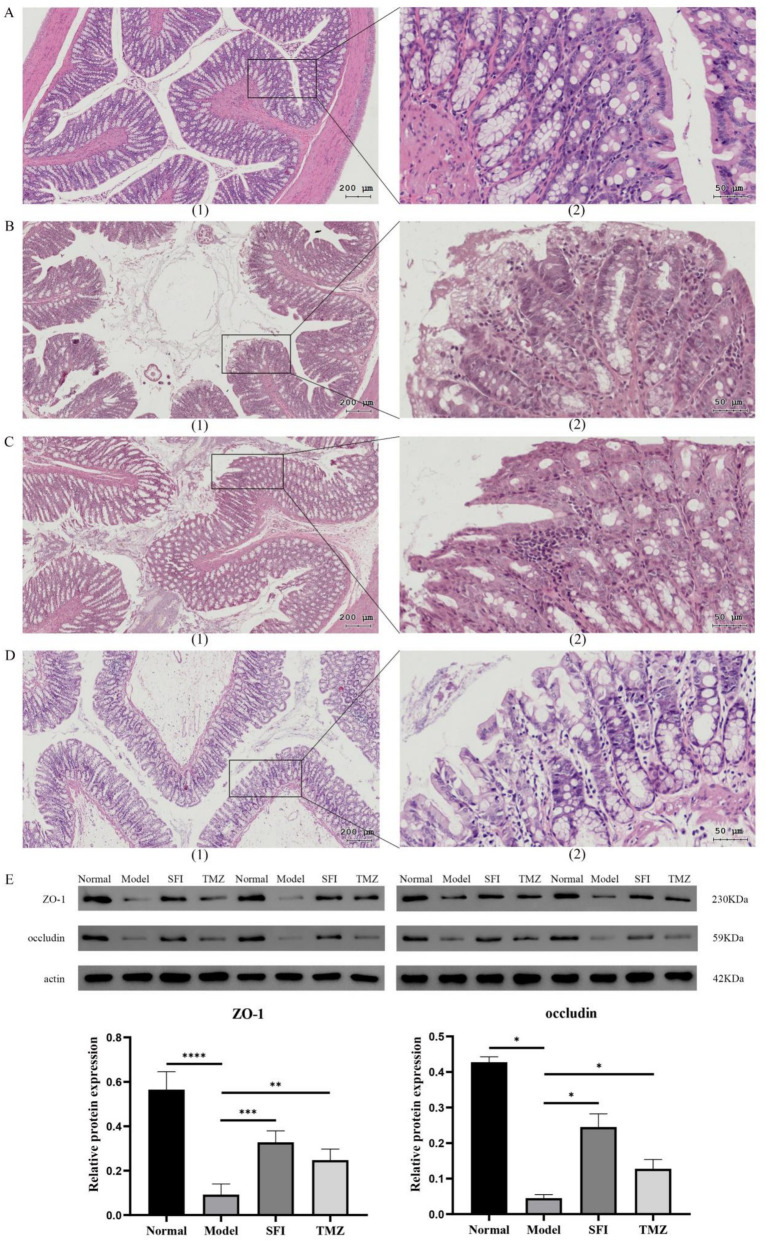
Colonic indices. **(A–D)** Colon tissue HE staining. **(A)** Normal group, **(B)** Model group, **(C)** SFI group, **(D)** TMZ group. (1)-The scale bar is 200 microns; (2)-The scale bar is 50 microns; **(E)** Western Blot. *p < 0.05; **p < 0.01; ***p < 0.001; ****p < 0.0001. Normal, Model, SFI and TMZ represent the blank control group, HF control group, Shenfu Injection treatment group and positive drug control group, respectively.

In the Normal group, the intestinal wall tissue structure was complete, the chorion was arranged neatly, multiple small glands were seen, and the acinar cells were closely arranged. Connective tissue fibers were evenly distributed and rich in collagen. There was no obvious inflammatory cell infiltration. In the Model group, the epithelial layer was arranged disorderly, and the villus epithelial exfoliation necrosis and inflammatory cell infiltration were observed. In some areas, the cells were different in size and irregular in shape, and the cytoplasm was condensed or vacuolated, suggesting that there might be abnormal cell proliferation or apoptosis. Increased pigmentation was seen in individual areas, probably due to pigmentation after tissue damage. Fibrous tissue proliferation was seen in some areas, suggesting possible fibrotic changes. In the SFI group, the epithelial layer was loosely arranged, the villous epithelium was not smooth, inflammatory cell infiltration was seen, and some areas of submucosa were exposed. Fibrosis appeared in some areas, manifested as increased collagen fibers. In the TMZ group, the epithelial cells were irregularly arranged, inflammatory cell infiltration was observed, and some epithelial cells were necrotic. Intraepithelial neoplasia (low-grade) was present and manifested as dysplasia of epithelial cells. Ulcers were formed in some areas, with mucosal defects, and granulation tissue hyperplasia was observed around the ulcers. Scarring and an increase in fibrous tissue were seen in some areas (Figures 4A–D).

Western Blot results indicate that compared to the Normal group, the expression of ZO-1 protein and occludin protein significantly decreased in the Model group. After treatment with Shenfu Injection and trimetazidine, the expression levels of these two proteins significantly increased. These results indicated that both Shenfu Injection and trimetazidine ameliorated the destruction of intestinal mucosal barrier function in ISO-induced HF rats (Figure 4E).

### Energy metabolism-related indices

**Figure 5:**
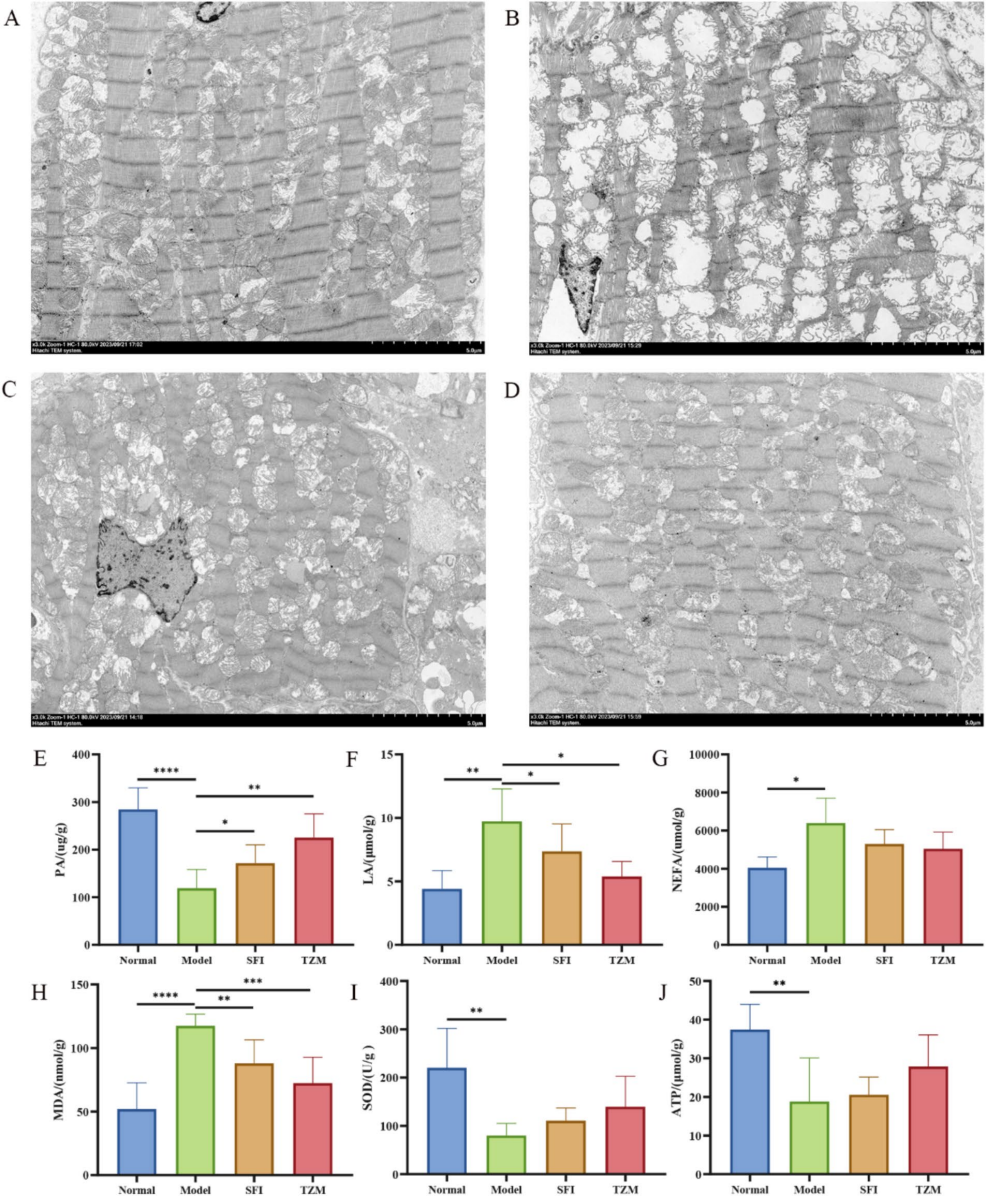
Transmission electron microscopy and biochemical detection. **(A–D)** Cardiac transmission electron microscope (TEM). **(A)** Normal group, **(B)** model group, **(C)** SFI group, **(D)** TMZ group; **(E)** Pyruvic acid, PA; **(F)** Lactic acid, LA; **(G)** Non-Esterified Fatty Acid, NEFA; **(H)** Malondialdehyde, MDA; **(I)** Superoxide Dismutase, SOD; **(J)** Adenosine Triphosphate, ATP. *p < 0.05; **p < 0.01; ***p < 0.001; ****p < 0.0001. Normal, Model, SFI and TMZ represent the blank control group, HF control group, Shenfu Injection treatment group and positive drug control group, respectively.

The results of heart transmission electron microscopy showed that the myofibrils in the Normal group were arranged neatly and the structure was dense, the morphology of mitochondria was normal, most of the membrane structure was complete, most of the cristae were arranged neatly and the structure was clear, the Z line and H band were clear and continuous. In the Model group, some myofibrils disappeared, most of the mitochondria were obviously swollen, the inner or outer membrane was ruptured, mitochondrial cribriform was dissolved and fractured, some of the mitochondria showed vacuolization of internal organization loss, and Z line and H band were broken. In the SFI group, the myofibrils were arranged disorderly, the mitochondria were not obviously swollen, the shape was regular, the cristae structure was clear and dense, and the Z line and H band were tortuous. The structure of myofibrils in the TMZ group was relatively normal, with a few mitochondria swelling, some cristae structures unclear, the inner and outer membranes relatively intact, and the Z line and H band clear and continuous (Figures 5A–D).

Biochemical analysis results showed that compared with the Normal group, the content of PA, SOD, and ATP in the Model group was significantly decreased, while the content of LA, NEFA, and MDA was significantly increased. After treatment, the PA content in the SFI group and TMZ group increased significantly, while the content of LA and MDA decreased significantly, and the content of NEFA, SOD, and ATP showed a trend of recovery (Figures 5E–J).

## High-throughput sequencing of 16S rRNA

### Quality control analysis of gut microbiota

**Figure 6:**
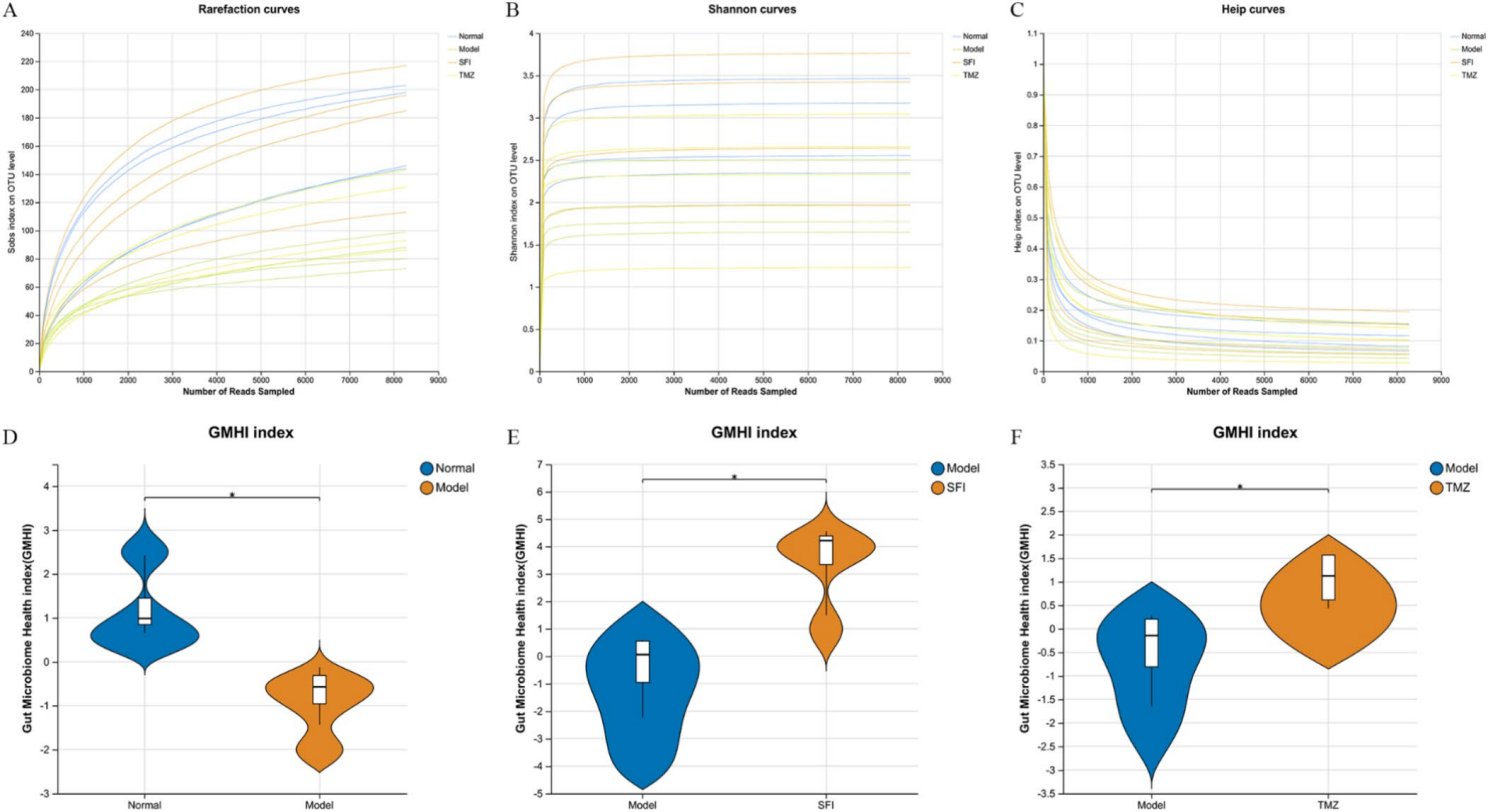
Rarefaction curves and microbiota health indices. **(A–C)** Rarefaction curves, **(A)** Sobs index, reflecting species richness; **(B)** Shannon index, reflecting species diversity; **(C)** Heip index, reflecting species evenness; **(D–F)** Gut Microbiome Health Index (GMHI). A robust index based on the species-level taxonomic features of gut microbiome samples to assess health status, focusing on determining the likelihood of disease, independent of clinical diagnosis; compared to alpha indices, the GMHI significantly outperforms ecological indicators in differentiating between healthy and non-healthy populations and shows strong reproducibility in stratification of healthy versus non-healthy groups, generally considered a marker of gut health and dysbiosis. *p < 0.05. Normal, Model, SFI, and TMZ represent the blank control group (*n* = 4), HF control group (*n* = 4), Shenfu Injection treatment group (*n* = 4), and positive drug control group (*n* = 4), respectively.

**Figure 7:**
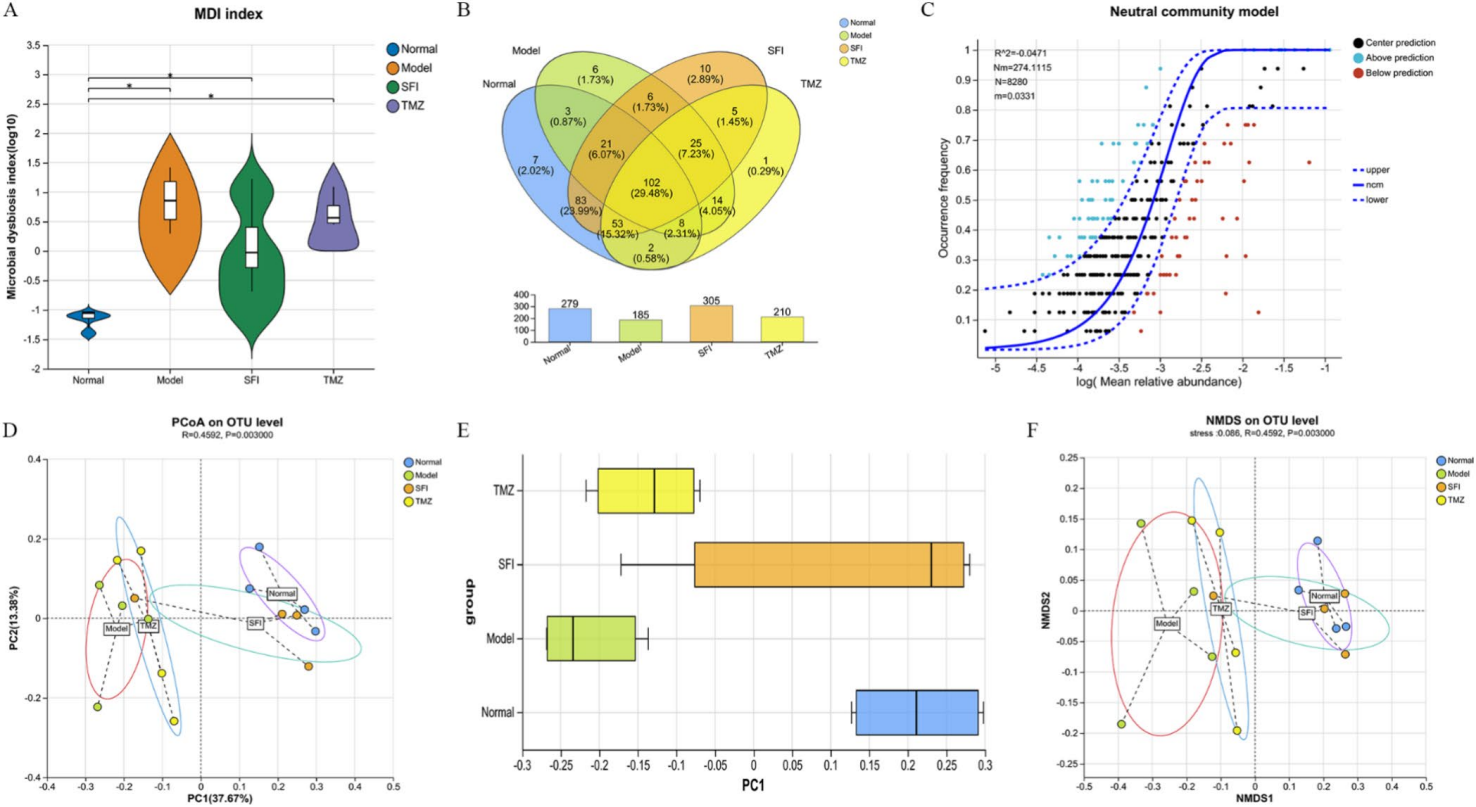
Bioinformatics analysis. **(A)** intestinal microbiota dysbiosis index (MDI); **(B)** Venn diagram. The numbers in the overlapping parts represent the number of species common to multiple groups, while the numbers in the non-overlapping parts represent the number of species unique to the corresponding group; **(C)** neutral community model (NCM). The x-axis represents the log value of the mean relative abundance of species [log (Mean relative abundance)], and the y-axis represents the predicted occurrence frequency (occurrence frequency); the solid line represents the fit of the neutral model, and the dashed lines above and below represent the 95% confidence level of the model’s prediction; R^2^ represents the overall goodness of fit of the neutral community model, with a higher R^2^ indicating closer alignment with the neutral model, meaning that the assembly of the community is more influenced by random processes and less influenced by deterministic processes. N describes the size of the metacommunity, which is the total abundance of all OTUs in each sample. m quantifies the migration rate at the community level, which is uniform for each community member (species-independent). A lower m value indicates more restricted species dispersal in the entire community, while a higher m value suggests less limitation on species dispersal. Nm is the product of the metacommunity size (N) and the migration rate (m) (Nm = N*m), quantifying the estimate of dispersal between communities and determining the correlation between occurrence frequency and regional relative abundance; **(D)** principal co-ordinates analysis (PCoA). Points of different colors or shapes represent samples from different groups. The closer two sample points are, the more similar their species composition is; **(E)** PCoA Box Plot The box plots in the graph represent the distribution dispersion of different group samples on the PC1 axis; **(F)** non-metric multidimensional scaling (NMDS). The closer two sample points are, the more similar their species composition is. Stress: It is used to test the quality of the NMDS analysis results. It is generally considered that when stress<0.2, the two-dimensional point plot of NMDS is representable and has some explanatory meaning; when stress<0.1, it can be considered a good sorting; when stress<0.05, it has very good representativeness. *p < 0.05. Normal, Model, SFI, and TMZ represent the blank control group (*n* = 4), HF control group (*n* = 4), Shenfu Injection treatment group (*n* = 4), and positive drug control group (*n* = 4), respectively.

A total of 130,960 valid sequences were obtained from 16 samples through sequencing, with an average of 8,185 quality-controlled valid sequences per sample. As the number of sequences increased, the sparse curve tended to flatten and extend to the right end of the x-axis, indicating that the current sequencing data volume was progressively reasonable and could be used for subsequent analysis (Figures 6A–C). The Neutral Community Model (NCM), developed by Sloan et al. (2006) is widely used in the analysis of microbial communities. To explore the factors influencing the assembly mechanisms of gut microbiota communities, this study utilized the NCM to analyze the ecological assembly of rat communities (Figure 7C). The low R^2^ value observed in the study (−0.0471) suggests that community construction deviates from the neutral model, implying that community formation is more driven by deterministic processes rather than random factors.

### Effects of Shenfu injection on the diversity of gut microbiota

A total of 346 OTUs were obtained by paired-end sequencing. The number of OTUs in the Normal group, Model group, SFI group, and TMZ group were 279, 185, 305, and 210, respectively (Figure 7B). This indicates that the number of OTUs in ISO-induced HF rats decreased, and the number of OTUs was restored after treatment with Shenfu Injection and trimetazidine. Additionally, the number of OTUs shared between the Normal group and the Model group was 134, the number of OTUs shared between the Normal group and the SFI group was 259, and the number of OTUs shared between the Normal group and the TMZ group was 165. This suggests that the microbial community structure of the SFI and TMZ groups gradually recovered after treatment, and their similarity to the Normal group’s microbial community was higher than that between the Model group and the Normal group.

**Figure 8:**
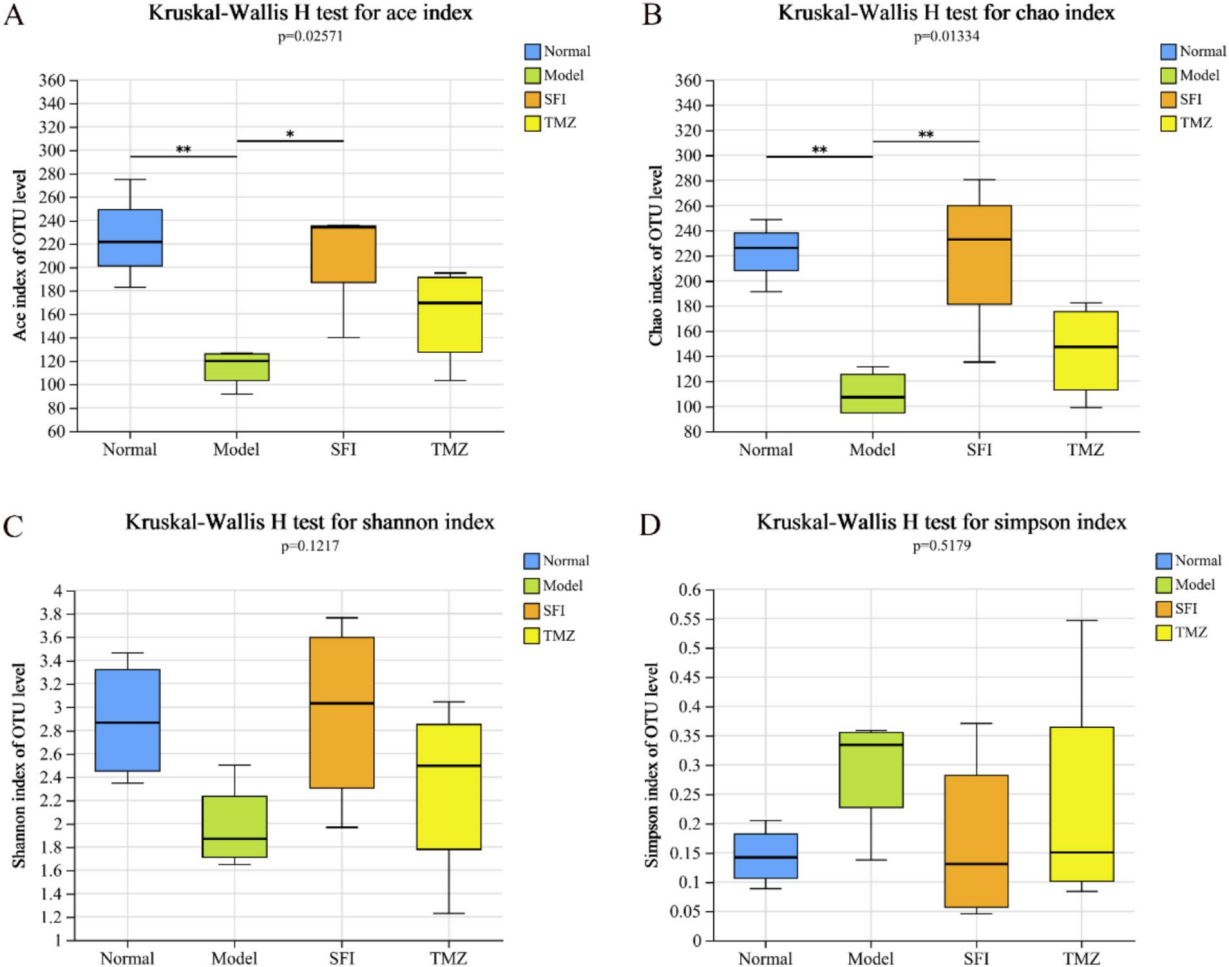
Alpha diversity index. **(A)** ACE index diagram; **(B)** Chao index diagram; **(C)** Shannon index diagram; **(D)** Simpson index diagram. *p < 0.05; **p < 0.01. Normal, Model, SFI and TMZ represent the blank control group (*n* = 4), HF control group (*n* = 4), Shenfu Injection treatment group (*n* = 4) and positive drug control group (*n* = 4), respectively.

Alpha diversity analysis is a key measure for assessing microbial diversity and richness (Figures 8A–D). Species richness is commonly quantified by the Chao and ACE indices, where higher index values indicate a greater richness of species; species diversity is evaluated by the Shannon and Simpson indices, with an increase in the Shannon index and a decrease in the Simpson index both indicating higher species diversity. In this experiment, the values of ACE, Chao, and Shannon in the Model group were all lower than those in the Normal group, while the Simpson value was higher than that in the Normal group. After treatment, in both the SFI and TMZ groups, the values of ACE, Chao, Shannon, and Simpson all showed a trend of recovery. The above results indicate that both Shenfu Injection and trimetazidine have a certain restorative effect on the richness and diversity of the gut microbiota in ISO-induced HF rats.

The results of GMHI and MDI jointly indicate that, compared to the Normal group, there is an imbalance in the intestinal ecology of the rats in the Model group. After treatment, the intestinal microenvironment of the SFI group and TMZ group has improved (Figures 6D–F, 7A).

### Effects of Shenfu injection on the gut microbiota structure

Beta diversity analysis is commonly used to evaluate the overall structure of microbial communities. Among these, PCoA is an unconstrained method for dimensionality reduction of data. In this experiment, we analyzed the differences in the gut microbiota among four groups of rats based on the Bray-Curtis distance algorithm and the ANOSIM test for inter-group differences (Figure 7D). In the total flora, the representativeness of PC1 and PC2 reached 37.67 and 13.38%, respectively. On the PC1 axis (Figure 7E), samples from the Normal group and the Model group were significantly separated, indicating that HF led to significant changes in the gut microbiota. After treatment, the distance between the TMZ group and the Model group was closer, suggesting that trimetazidine had a weaker effect on repairing the structure of the gut microbiota in HF rats; whereas the distance between the SFI group and the Model group gradually increased, and the distance between the SFI group and the Normal group gradually decreased. Therefore, it can be inferred that Shenfu Injection surpasses trimetazidine in promoting the recovery of the gut microbiota structure in HF rats.

Additionally, as another method for Beta diversity analysis, NMDS analysis is a data analysis method that simplifies research samples in multidimensional space to a lower-dimensional space for positioning, analysis, and classification, while retaining the original relationships between samples (Figure 7F). Based on the NMDS1 axis, the samples from left to right are the Model group, TMZ group, SFI group, and Normal group, indicating that in the comparison of similarity with the Normal group samples, the least similar are the Model group samples, followed by the TMZ group samples, and the most similar are the SFI group samples. These results suggest that trimetazidine has a certain restorative effect on the changes in microbial community structure induced by HF, but the effect is weaker than that of Shenfu Injection.

### Species composition analysis

**Figure 9:**
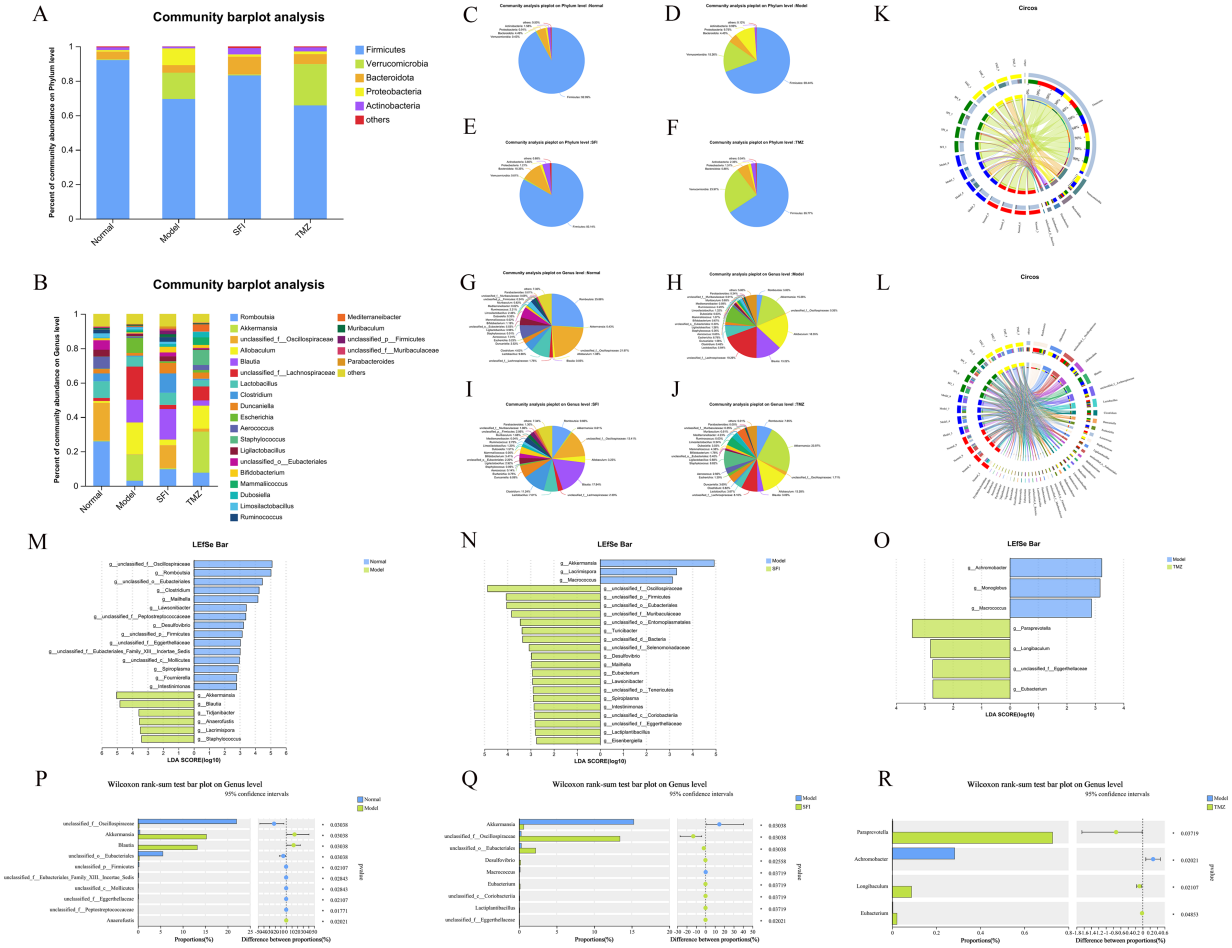
Species composition and differential analysis. **(A,B)** Community bar plots. These represent the bacterial composition at the phylum and genus levels, respectively. The vertical axis indicates the proportion of each species within the sample, with different colored bars representing different species. The length of the bars corresponds to the size of the proportion that each species occupies. **(C–J)** Community pie charts. Each pie chart represents the microbial community composition of a specific group at the selected taxonomic level. Different colors denote different species, and the area of the pie slices represents the percentage of each species. **(K,L)** Circos sample-species relationship diagrams. These visualizations use circular diagrams to depict the corresponding relationships between species and samples, aiding in understanding the distribution proportions of species among different groups and also showing the types of microorganisms present in each group along with their relative abundances. **(M–O)** LDA discriminant bar plots. These statistically represent microbial groups with significant effects across multiple groups. LDA scores are obtained through LDA analysis, with higher LDA scores indicating a greater impact of species abundance on the differential effect. **(P–R)** Two-group difference tests. The X-axis represents different groups, with different colored boxes indicating different groups. The Y-axis represents the average relative abundance of a particular species across different groups. *p < 0.05; **p < 0.01. Normal, Model, SFI, and TMZ represent the blank control group (*n* = 4), HF control group (*n* = 4), Shenfu Injection treatment group (*n* = 4), and positive drug control group (*n* = 4), respectively.

The bar chart of community composition displays the types and relative abundance of the gut microbiota, allowing for a clear visualization of the variation trends of species across different groups (Figures 9A,B). The pie charts present the specific values of the relative abundance of the gut microbiota (Figure 9C–J). The visualization of the species-sample relationships is shown in the Circos plots (Figures 9K–L).

### Composition changes of the gut microbiota at the phylum level

At the phylum level, five of the most abundant bacteria were detected, namely Firmicutes, Verrucomicrobia, Bacteroidota, Proteobacteria, and Actinobacteria, all with relative abundance above 1% (Figure 9A). The relative abundance of Firmicutes was Normal group > SFI group > Model group > TMZ group, accounting for 92.09, 83.14, 69.44, 65.77%, respectively. The relative abundance of Verrucomicrobia was TMZ group > Model group > SFI group > Normal group, accounting for 23.97, 15.26, 0.61 and 0.43%, respectively. The relative abundance of Bacteroidota was in the order of SFI group > TMZ group > Normal group > Model group, accounting for 10.35, 5.86, 4.49 and 4.43%, respectively. The relative abundance of Proteobacteria was Model group > TMZ group > SFI group > Normal group, accounting for 9.75, 1.51, 1.21 and 0.91%, respectively. The relative abundance of Actinobacteria was SFI group > TMZ group > Normal group > Model group, accounting for 3.80, 2.35, 1.58, 0.99%, respectively.

### Composition changes of the gut microbiota at the genus level

At the genus level, a total of 24 bacterial species were detected, with those accounting for <1% of the total classified as “others” (Figure 9B). The relative abundance of bacteria in the Normal group, from high to low, was as follows: Romboutsia 25.86%, unclassified_f__Oscillospiraceae 21.97%, Lactobacillus 9.66%, Aerococcus 7.01%, unclassified_o__Eubacteriales 5.53%, Clostridium 4.63%, etc. In the Model group, the relative abundance was unclassified_f__Lachnospiraceae 19.29%, Allobaculum 18.35%, Akkermansia 15.26%, Blautia 13.22%, Escherichia 8.79%, Lactobacillus 5.84%, etc. For the SFI group, the relative abundance from high to low was Blautia 17.84%, unclassified_f__Oscillospiraceae 13.41%, Clostridium 11.24%, Romboutsia 9.66%, Lactobacillus 7.01%, Duncaniella 6.09%, etc. In the TMZ group, the relative abundance was Akkermansia 23.97%, Allobaculum 13.28%, Staphylococcus 8.82%, unclassified_f__Lachnospiraceae 8.10%, Romboutsia 7.65%, Mammaliicoccus 4.38%, etc.

### Species difference analysis

Through linear regression analysis, key species that may play a critical role in the occurrence and progression of HF were identified at the genus level. Compared with the Normal group, bacteria such as Akkermansia, Blautia, Tidjanibacter, Anaerofustis, Lacrimispora, and Staphylococcus were significantly enriched in the Model group. The abundance of bacteria including unclassified_f__Oscillospiraceae, Romboutsia, unclassified_o__Eubacteriales, Clostridium, Mailhella, and Lawsonibacter significantly decreased in the Model group. In contrast to the Model group, bacteria like unclassified_f__Oscillospiraceae, unclassified_p__Firmicutes, unclassified_o__Eubacteriales, unclassified_f__Muribaculaceae, unclassified_o__Entomoplasmatales, and Turicibacter were significantly enriched in the SFI group, while the abundance of Akkermansia, Lacrimispora, and Macrococcus significantly decreased in the SFI group. Compared with the Model group, bacteria such as Paraprevotella, Longibaculum, unclassified_f__Eggerthellaceae, and Eubacterium were significantly enriched in the TMZ group, and the abundance of Achromobacter, Monoglobus, and Macrococcus significantly decreased in the TMZ group (Figures 9M–O).

Analysis of intergroup differences at the genus level revealed significant differences in the abundance of bacterial genera such as unclassified_f__Oscillospiraceae, Akkermansia, Blautia, unclassified_o__Eubacteriales, and unclassified_p__Firmicutes between the Normal and Model groups. Significant differences in the abundance of bacterial genera including Akkermansia, unclassified_f__Oscillospiraceae, unclassified_o__Eubacteriales, Desulfovibrio, and Macrococcus were observed between the Model and SFI groups. Additionally, significant differences in the abundance of bacterial genera such as Paraprevotella, Achromobacter, Longibaculum, and Eubacterium were found between the Model and TMZ groups (Figures 9P–R).

## Targeted metabolomics profiling of SCFAs

### Standards and samples TIC

**Figure 10:**
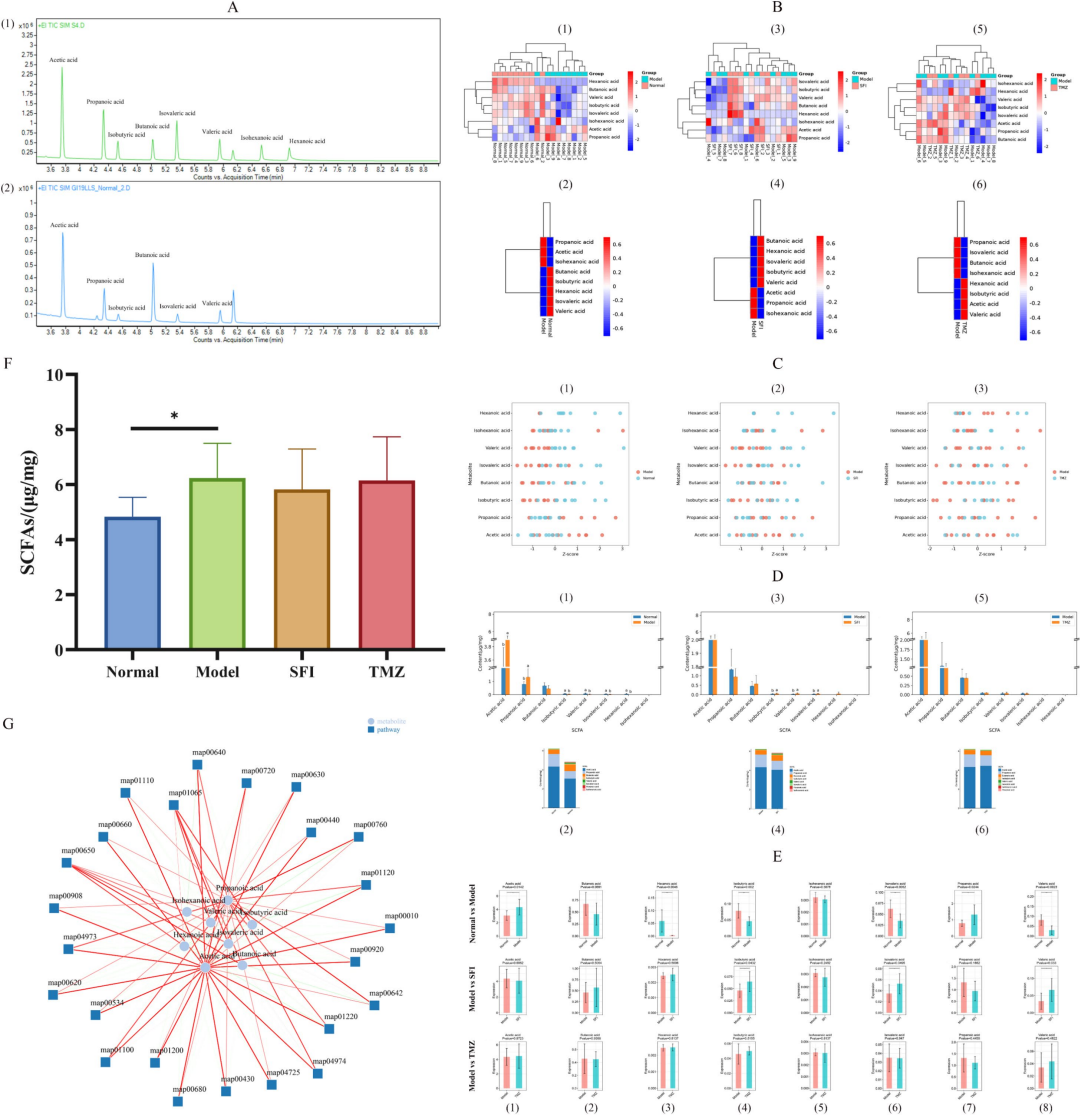
Targeted metabolomics analysis of SCFAs. **(A)** TIC (1)—standard TIC; (2)—sample TIC; **(B)** Cluster analysis of expression patterns. Each column in the figure represents a sample/group, each row represents a SCFA, and the color in the figure indicates the relative expression level of SCFAs within that sample/group. The left side shows a dendrogram of SCFA clustering, and the right side lists the names of SCFAs. The closer two SCFA branches are, the more similar their expression levels are. The top of the figure depicts a dendrogram of sample clustering, with different color blocks representing different samples/groups, and the bottom lists the names of the samples/groups. The closer the clustering branches of two samples/groups are, the more similar the expression patterns of all SCFAs in the two samples/groups, indicating a similar trend in the changes of SCFA expression levels. (1, 3, 5)—Heatmaps of expression pattern clustering for all samples; (2, 4, 6)—Heatmaps of expression pattern clustering for all groups; (1, 2)—Normal vs. Model, (3, 4)—Model vs. SFI, (5, 6)—Model vs. TMZ. **(C)** Metabolite Z-score Plot. The x-axis represents Z-score values, which are used to measure the relative abundance of metabolites at the same level, while the y-axis lists the names of metabolites. Each dot represents a sample, and the color of the dot indicates the different groups. (1)—Normal vs. Model, (2)—Model vs. SFI, (3)—Model vs. TMZ. **(D)** Content Distribution Analysis. (1, 3, 5)—Histograms of content distribution the Figure displays the content of 8 SCFA indicators across different groups. The x-axis represents SCFAs, the y-axis represents content, and the bars of different colors represent different groups. Additionally, the significance of differences in SCFAs among groups was calculated by ANOVA and *post-hoc* multiple comparisons. For the same indicator, if the letters assigned to two groups are the same, it indicates no significant difference in content between the two groups. If the letters are different, it indicates a significant difference in content between the two groups. If no letter is present, no difference is indicated. (2, 4, 6)—Relative abundance histograms the figure shows the relative abundance of 8 SCFAs in each group, calculated as a proportion, and the average values are plotted as histograms. The x-axis represents the group, the y-axis represents content, and different colors represent different SCFAs. (1, 2)—Normal vs. Model, (3, 4)—Model vs. SFI, (5, 6)—Model vs. TMZ. **(E)** Content box plots. (1–8) correspond to Acetic acid, Butanoic acid, Hexanoic acid, Isobutyric acid, Isohexanoic acid, Isovaleric acid, Propanoic acid, and Valeric acid, respectively. **(F)** SCFAs, Short chain fatty acids. **(G)** Metabolite pathway-associated network diagram. The lines represent the correlation between entities; the darker the line, the stronger the correlation. Green indicates a negative correlation, and red indicates a positive correlation. The thicker the line, the smaller the *p*-value. *p < 0.05; **p < 0.01; ***p < 0.001. Normal, Model, SFI, and TMZ represent the blank control group (*n* = 9), HF control group (*n* = 9), Shenfu Injection treatment group (*n* = 8), and positive drug control group (*n* = 6), respectively.

This experiment analyzed eight types of SCFAs in a span of 9 min. TIC exhibits superior separation and well-pronounced peak formations for each compound analyzed (Figure 10A).

### Cluster analysis of expression patterns

Compared to the Normal group, the relative expression levels of Butanoic acid, Isobutyric acid, Hexanoic acid, Isovaleric acid, and Valeric acid in the Model group were decreased, whereas the relative expression levels of Propanoic acid, Acetic acid, and Isohexanoic acid were increased. In contrast to the Model group, the SFI group exhibited elevated relative expression levels of Butanoic acid, Hexanoic acid, Isovaleric acid, Isobutyric acid, and Valeric acid, with reduced relative expression levels of Acetic acid, Propanoic acid, and Isohexanoic acid. Following treatment, the TMZ group, compared to the Model group, showed increased relative expression levels of Hexanoic acid, Isobutyric acid, Acetic acid, and Valeric acid, while the relative expression levels of Propanoic acid, Isovaleric acid, Butanoic acid, and Isohexanoic acid were decreased (Figure 10B).

To present a clearer comparison of the relative content of metabolites at the same level, Z-score analysis was employed (Figure 10C).

### Content distribution analysis

To visually represent the proportion of various SCFAs, we constructed a histogram depicting the content distribution of SCFAs (Figure 10D). It can be observed that the top three SCFAs with the highest content within each group are Acetic acid, Propanoic acid, and Butanoic acid, respectively.

### Content analysis of each index

Through pairwise comparisons between groups, we found significant differences in the concentrations of Acetic acid, Hexanoic acid, Isobutyric acid, Isovaleric acid, Propanoic acid, and Valeric acid between the Normal group and the Model group. The concentrations of Isobutyric acid, Isovaleric acid, and Valeric acid in the SFI group showed a significant trend of regression when compared to the Model group. Unfortunately, no significant changes in SCFAs were observed in the TMZ group when compared to the Model group (Figure 10E).

### Differential analysis of the cumulative content of SCFAs

Comparatively, the Model group exhibited a significant increase in the concentration of SCFAs when contrasted with the Normal group, whereas both the SFI and TMZ groups demonstrated a trend of decrease in SCFAs content (Figure 10C).

### Metabolite pathway-associated network

SCFAs can influence pathways primarily involving biochemical processes related to energy metabolism, including carbohydrate metabolism, amino acid metabolism, lipid metabolism, cofactor and vitamin metabolism. The affected pathways include: map00650: Acetate Metabolism, map04725: Butanoate Metabolism, map00680: Caprolactam Degradation, map04973: Valine, Leucine and Isoleucine Degradation, map01120: Glycerophospholipid Metabolism, map00620: Pyruvate Metabolism, map01220: Citric Acid Cycle (TCA Cycle), map00660: Glyoxylate and Dicarboxylate Metabolism, map00630: Glycolysis/Gluconeogenesis, map00920: Selenocompound Metabolism, map01100: Secondary Bile Acid Biosynthesis, map01200: Carbon Fixation in Photosynthetic Organisms, map04974: Tryptophan Metabolism, map00430: Nitrogen Metabolism, map00640: Pyrimidine Metabolism, map00642: Pyrimidine Metabolism, map00720: Lysine Biosynthesis, map00760: Urea Cycle, map00440: Porphyrin and Chlorophyll Metabolism, map01110: Fatty Acid Degradation, map00908: Folate Biosynthesis, map01065: Benzoate Degradation via CoA, map00534: Fatty Acid Elongation, map00010: Glycolysis/Gluconeogenesis.

### Clinical factor correlation analysis and diagnostic model construction

**Figure 11:**
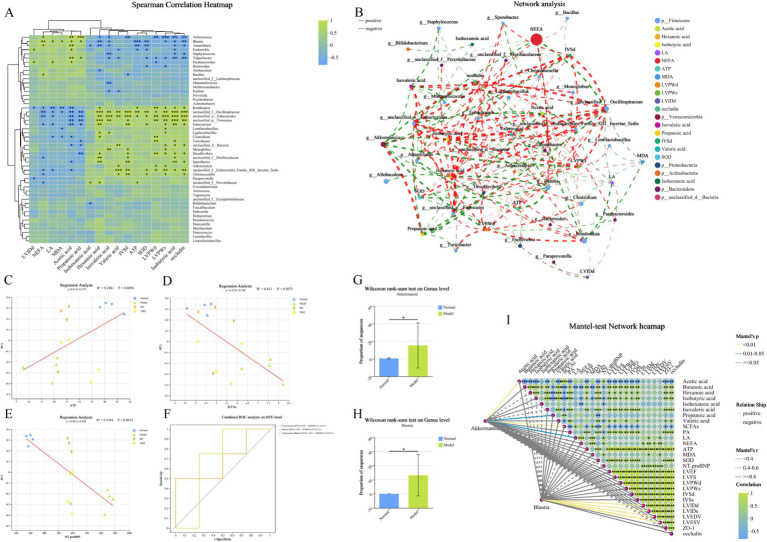
Clinical factor correlation analysis and diagnostic model construction. **(A)** Heatmap of the correlation between genus-level bacteria and clinical indicators. The X-axis and Y-axis represent clinical factors and species, respectively. The correlation R values and *p* values are calculated. The R values are displayed in different colors in the figure, with the color range of different R values indicated in the legend on the right. The clustering trees of species and clinical factors are presented on the left and top sides, respectively. **(B)** Network analysis. The correlation network diagram illustrates the relationships between the top 50 species (bacteria color indicates Phylum level) in total abundance and clinical factors (correlation coefficient type: Spearman; absolute value of correlation coefficient ≥ 0.5; *p* < 0.05). The size of the nodes in the diagram reflects the abundance of species/clinical factor levels, with different colors representing different species/clinical factors. The color of the connecting lines indicates positive or negative correlations, with red for positive and green for negative. The thickness of the lines represents the magnitude of the correlation coefficient; the thicker the line, the higher the correlation between species. The more lines, the closer the connection between the nodes. **(C–E)** Ordination regression analysis. The X-axis represents clinical factors, and the Y-axis represents the Beta diversity (PCoA, bray_curtis) ordination axis. R^2^ is the coefficient of determination, representing the proportion of variation explained by the regression line. A higher R^2^ indicates that the clinical factor explains a greater degree of the differences in samples along the ordination axis. **(F)** Integrated ROC analysis. The X-axis is 1-Specificity, ranging from 0 to 1; the Y-axis is Sensitivity, also ranging from 0 to 1. The AUC indicated in the figure is the area under the corresponding curve. The AUC values typically range between 1.0 and 0.5. When AUC > 0.5, the closer the AUC is to 1, the better the diagnostic performance. **(G,H)** Comparison of the abundance of Akkermansia and Blautia bacteria in the Normal and Model groups. **(I)** Mantel Test Network Heatmap. The lines in the figure represent the correlation between the community and clinical factors, while the heatmap represents the correlation between clinical factors. The thickness of the lines indicates the magnitude of the correlation between the community and clinical factors, drawn using Mantel’s r (absolute value of R). Relationship: Positive and Negative indicate the positive and negative correlations between the community and environmental factors. Different colors in the heatmap represent positive and negative correlations, with the depth of color indicating the magnitude of the correlation. Asterisks in the color blocks represent significance. *p < 0.05; **p < 0.01; ***p < 0.001. Normal, Model, SFI, and TMZ represent the blank control group, HF control group, Shenfu Injection treatment group, and positive drug control group, respectively.

Variance Inflation Factor (VIF) values exceeding 10 for the related indicators are considered to affect subsequent correlation analysis due to the presence of multicollinearity among clinical factors. After screening, the related indicators with VIF values less than 10 include Acetic acid, Hexanoic acid, Isobutyric acid, Isohexanoic acid, Isovaleric acid, Propanoic acid, Valeric acid, LA, NEFA, ATP, MDA, SOD, LVPWd, LVPWs, IVSd, LVIDd, and occludin, with respective VIF values of 5.41, 2.23, 5.60, 1.40, 3.23, 3.45, 3.56, 8.13, 7.56, 6.33, 7.25, 7.93, 2.91, 4.02, 2.33, 1.23, and 5.13. The correlation heatmap illustrates the Spearman correlation coefficients between the top 50 dominant microbial genera (at the genus level) in the rat gut and clinical factors (Figure 11A). The results indicate that six bacterial genera, Akkermansia, Blautia, unclassified_f__Oscillospiraceae, unclassified_o__Eubacteriales, unclassified_p__Firmicutes, and unclassified_f__Eubacteriales_Family_XIII._Incertae_Sedis, exhibit strong correlations with certain clinical factors (*p* < 0.001). Detailed information on the correlation R values and *p* values between bacteria and clinical factors can be found in the Supplementary Tables 4, 5. Furthermore, a bipartite correlation network diagram between species and clinical factors was constructed (Figure 11B), with detailed information in the Supplementary Tables 6, 7. Considering the species difference analysis (Figures 9M–R) and the results of the aforementioned correlation analysis, we selected two classified bacterial genera (Akkermansia and Blautia) with significant abundance differences (Figures 11G,H) and strong correlations with clinical factors for subsequent analysis. Subsequently, a joint ROC analysis was conducted on the OTU sets of Gut microbiota (Akkermansia and Blautia) and SCFAs data (Acetic acid, Hexanoic acid, Isobutyric acid, Isovaleric acid, Propanoic acid, Valeric acid, etc., six SCFAs with significant differences between the Normal and Model groups) to evaluate their diagnostic capability for the disease. The results showed that the combination of Gut microbiota and SCFAs had an accuracy rate of 69% for disease diagnosis (Figure 11C), which is relatively weak, possibly due to the small number of samples within groups and the limited number of classified bacteria with significant intergroup differences and strong correlations with clinical factors based on 16S rRNA high-throughput sequencing. Finally, a Mantel Test network heatmap was created based on the two bacteria, Akkermansia and Blautia, to further visualize the associations between Gut microbiota and clinical factors, as well as the associations among clinical factors (Figure 11I), with detailed information in the Supplementary Tables 8, 9. Additionally, a regression analysis was performed to study the correlation between ATP, SCFAs, and the structure of the gut microbial community to assess the impact of ATP, SCFAs, and NT-proBNP on the differences in community composition (Figures 11C–E). The results indicate that ATP is significantly correlated with Gut microbiota (*p* = 0.0496) and explains 24.81% of the variation; SCFAs are also significantly correlated with Gut microbiota (*p* = 0.0073) and explain 41.30% of the variation; NT-proBNP is significantly correlated with Gut microbiota (*p* = 0.0012) and explains 53.94% of the variation.

## Discussion

**Figure 12:**
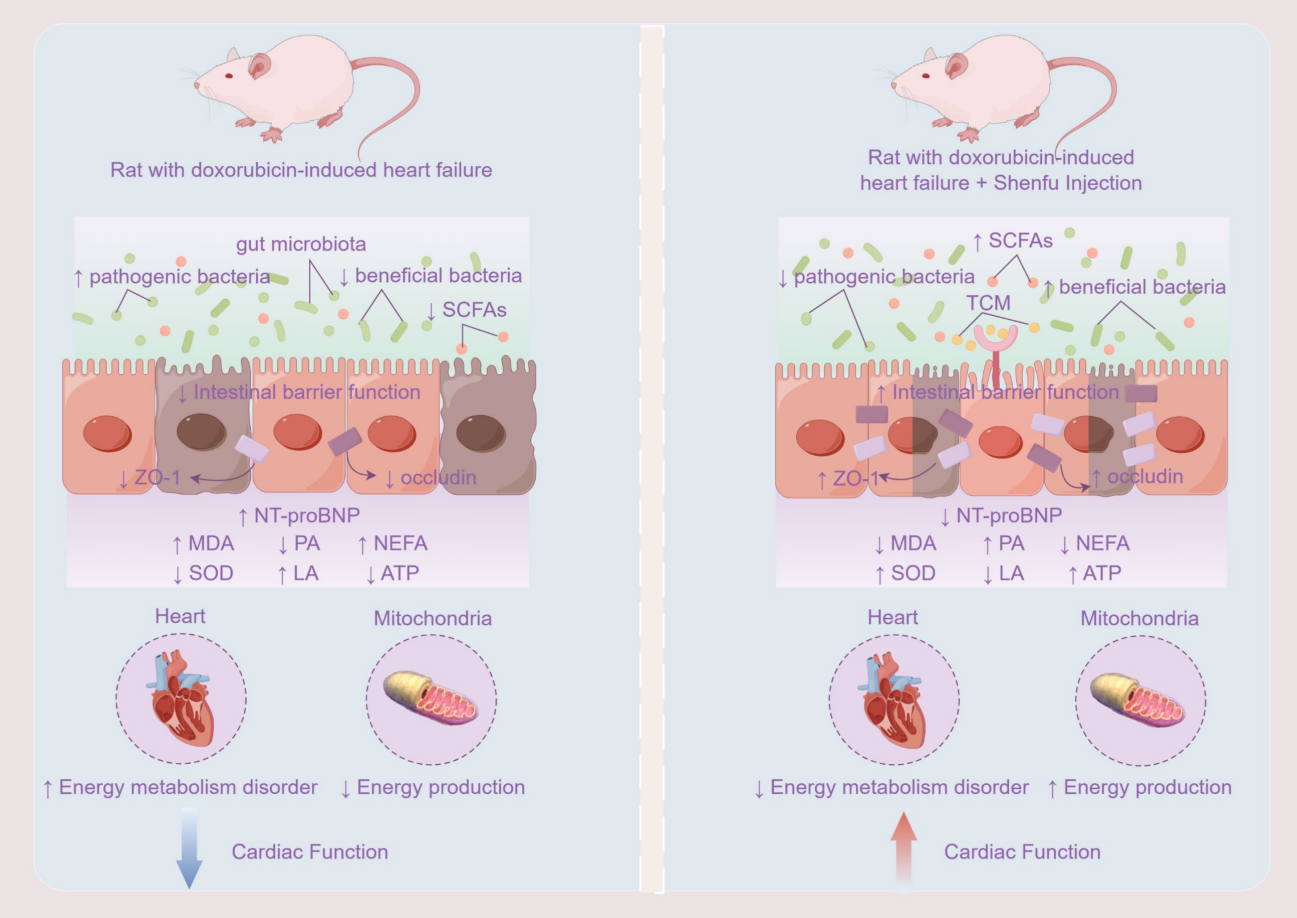
“Heart-gut” communication mechanism diagram (this figure was drawn using Figdraw 2.0).

The “Heart-Gut” communication mechanism diagram for this study is shown in Figure 12. The heart is a high-energy-consuming organ, with 90% of the energy required by normal adult myocardium provided by mitochondria (He et al., 2021). As the primary site for energy production in cardiomyocytes, healthy myocardial mitochondria can flexibly utilize various energy substrates, with mitochondrial oxidation of fatty acids accounting for 60–90% of mitochondrial energy production, representing the main energy source for the myocardium. The remaining 10–40% is produced by glucose metabolism, and a minimal portion comes from amino acids and ketone bodies. In failing hearts, an energy “metabolic remodeling” occurs, which is considered an important hallmark of HF (van Bilsen et al., 2004; Huang et al., 2020). During HF, the oxygen supply to cardiomyocytes is reduced, leading to a shift in myocardial energy metabolism patterns to meet the energy demands of myocardial activity, characterized by a decrease in fatty acid aerobic oxidation and an enhancement of glucose anaerobic glycolysis (Tung et al., 2022). In the terminal stage of HF, severe hypoxia leads to insulin resistance in the myocardium, reducing glucose uptake; simultaneously, weakened myocardial fatty acid oxidation capacity results in the accumulation of fatty acids within cardiomyocytes, causing fatty acid accumulation toxicity, triggering mitochondrial oxidative stress responses, leading to mitochondrial structural damage and dysfunction, insufficient energy supply to cardiomyocytes, and exacerbating the progression of HF (Turer et al., 2010). The results of this experiment confirmed that compared to healthy rats, the content of PA in the myocardium of HF rats induced by doxorubicin significantly decreased, while the contents of LA and NEFA significantly increased, suggesting a shift in myocardial energy metabolism substrates from fatty acids to glucose, with enhanced anaerobic glycolysis of glucose in HF rats. The decrease in ATP content in the myocardium of HF rats, along with decreased SOD content and increased MDA content, suggests that the insufficient energy supply in the myocardium of HF rats may be related to mitochondrial damage caused by oxidative stress, which is confirmed by the results of transmission electron microscopy. Trimetazidine, as a metabolic cardioprotective agent, can improve the energy metabolism rate of cardiomyocytes and reduce cell damage by promoting ATP production through the glucose metabolism process while decreasing LA production and oxygen consumption, and its efficacy has been confirmed in this experiment. Shenfu Injection has also been found to improve cardiac function by regulating myocardial energy metabolism. Compared to untreated HF rats, HF rats treated with Shenfu Injection showed significant improvements in ATP, PA, LA, NEFA, MDA, SOD, and mitochondrial morphology gradually recovered.

In recent years, an increasing number of studies have elucidated that the gut microbiota plays a significant role in the onset and progression of CVDs (Zou et al., 2022; Wang et al., 2011; He et al., 2024). During HF, the weakened cardiac pumping function leads to a slowing of systemic blood circulation, consequently reducing blood perfusion in the intestines, which can compromise the integrity of the intestinal mucosa and impair the gut barrier function. The intestinal mucosa serves as the primary habitat for gut microbiota, and a symbiotic relationship exists between the microbiota and the intestinal mucosa. When the mucosal layer is damaged, the balance of the gut microbiota may be disrupted. To validate this hypothesis, we employed HE staining, WB assays, and 16S rRNA high-throughput sequencing to uncover that, compared to healthy rats, the myocardial and colonic tissues of doxorubicin-induced HF rats exhibited significant pathological alterations. The expression of ZO-1 and occludin proteins, which reflect the integrity of the intestinal barrier, significantly decreased, the gut microbiota health index markedly declined, the dysbiosis index significantly increased, and the number of OTUs decreased. Alpha diversity indices indicated a significant reduction in species richness and a decrease in species diversity within the gut microbiota of rats with HF. Beta diversity analysis revealed a marked alteration in the structure of the gut microbiota of HF rats compared to healthy controls. These findings corroborated the aforementioned analyses. Following Shenfu Injection treatment, as compared to untreated HF rats, the SFI group exhibited mitigated histological lesions in myocardial and colonic tissues, a significant increase in ZO-1 and occludin protein expression, a notable rise in the gut microbiota health index, a decrease in the dysbiosis index, an increase in OTUs, a significant increase in species richness, an enhancement of species diversity, and an improved microbiota structure. After trimetazidine treatment, as compared to untreated HF rats, the TMZ group showed a certain degree of improvement in myocardial and colonic tissue lesions, a significant increase in ZO-1 and occludin protein expression, a notable rise in the gut microbiota health index, a decrease in the dysbiosis index, an increase in OTUs, and a recovery of species richness and diversity, though the improvement in microbiota structure was not significant. Intergroup difference analysis revealed that, compared to the Normal group, the Model group exhibited a significant reduction in the abundance of several recognized beneficial bacteria, such as Romboutsia (Zhao et al., 2024), Clostridium (Yoon et al., 2024), Mailhella (Shao et al., 2023), Lawsonibacter (Yau et al., 2023), and Fournierella (Yan et al., 2022), with exceptions including Akkermansia (Zhao et al., 2023) and Blautia (Li et al., 2024). The abundance of conditional pathobionts like Anaerofustis (Feng et al., 2022) significantly increased, whereas the abundance of Desulfovibrio (Song et al., 2018) and Intestinimonas (Bui et al., 2015) significantly decreased. Spiroplasma, Tidjanibacter, and Lacrimispora were found to be significantly altered in the intestinal tract of HF rats for the first time, with Spiroplasma abundance significantly decreasing and Tidjanibacter and Lacrimispora abundance significantly increasing in the Model group. Post-treatment, the SFI group saw a significant rebound in the abundance of Akkermansia, Lacrimispora, Desulfovibrio, Mailhella, Lawsonibacter, Spiroplasma, and Intestinimonas, whereas the TMZ group did not exhibit a marked rebound in bacterial abundance changes due to HF. Moreover, certain bacteria that did not show significant changes between the Normal and Model groups exhibited marked alterations within the SFI and TMZ groups, suggesting potential pharmacological impacts on the gut microbiota. In summary, both Shenfu Injection and trimetazidine can enhance cardiac function by optimizing energy metabolism; however, Shenfu Injection demonstrates more pronounced effects in regulating the gut microbiota and improving intestinal health compared to trimetazidine.

HF, as the end-stage of cardiovascular disease, is characterized by myocardial remodeling, myocardial injury, and disordered energy metabolism as its primary pathophysiological features. The gut microbiota may participate in the occurrence and development of HF by influencing these pathophysiological processes. SCFAs, as one of the main metabolic products of the gut microbiota, are produced by the fermentation of dietary fiber by gut bacteria and play multiple important physiological roles in the intestinal tract, including providing energy for intestinal cells. Previous studies have shown that SCFAs produced by the gut microbiota are beneficial to cardiac health, such as by improving lipid metabolism, reducing inflammation, and alleviating oxidative stress. However, evidence regarding their ability to provide energy for myocardial cells is limited. Utilizing targeted metabolomics technology, we observed that the total SCFAs content in the feces of doxorubicin-induced HF rats was significantly higher than that in healthy rats, with significant differences in the content of Acetic acid, Hexanoic acid, Isobutyric acid, Isovaleric acid, Propanoic acid, and Valeric acid. Pathway correlation analysis further indicated that these SCFAs are closely related to certain energy metabolism pathways. Ordinal regression analysis revealed a significant correlation between SCFAs and ATP content with the Gut microbiota. These findings suggest that the gut microbiota may play a compensatory role during HF, attempting to provide an additional energy source by promoting the production of SCFAs, thereby potentially supporting myocardial cell function and alleviating symptoms of HF. After treatment with Shenfu Injection, the SCFAs content in HF rats was restored, whereas the changes in SCFAs content in HF rats treated with trimetazidine were not significant.

To uncover the bacterial species that play a pivotal role in the development of HF, we identified two bacterial genera that exhibited significant differences between healthy rats and doxorubicin-induced HF rats, and were strongly associated with clinical factors: Akkermansia and Blautia. Akkermansia belongs to the phylum Verrucomicrobia. Further investigation revealed that the sole known member of Akkermansia is *Akkermansia muciniphila* (*A. muciniphila*), first discovered by Derrien et al. (2008) and Karcher et al. (2021). As a common microbial symbiont in humans and animals, Akkermansia has been demonstrated to confer numerous benefits, including the improvement of the intestinal mucosal barrier function, the maintenance of glucose and lipid metabolism balance, and the exertion of anti-inflammatory effects (Cani et al., 2022). *A. muciniphila* is considered a next-generation probiotic with promising clinical applications. A plethora of studies have indicated a strong association between *A. muciniphila* and a variety of metabolic disorders, including obesity, diabetes, CVDs, and non-alcoholic fatty liver disease (Niu H. et al., 2024; Yan et al., 2021; Dao et al., 2016). The potential benefits of Akkermansia/*A. muciniphila* derive from its ability to degrade mucins in the intestinal mucus layer and utilize them as the sole carbon and nitrogen source to produce SCFAs, predominantly acetic acid and propionic acid (Kim et al., 2021; Li Z. et al., 2021; de Vos, 2017). These SCFAs exert their biological effects both in the intestinal and extraintestinal environments by activating G protein-coupled receptors, inhibiting histone deacetylase activity, and modulating cellular metabolism (Pluznick et al., 2013; Kaisar et al., 2017; Schulthess et al., 2019). Studies have shown that acetic acid can stimulate GPR43 in white adipose tissue (WAT) to improve glycolipid metabolism (Kimura et al., 2013). Propionic acid, before reaching the liver, can be converted into glucose via intestinal gluconeogenesis, thereby reducing hepatic gluconeogenesis and conferring metabolic benefits that promote energy homeostasis (De Vadder et al., 2014; Anderson and Bridges, 1984). Additionally, Lukovac et al. (2014) discovered that many transcription factors regulating fat metabolism and proliferation are simultaneously influenced by *A. muciniphila* and propionic acid. These findings suggest that SCFAs have a regulatory role in energy metabolism. In our experiment, Spearman correlation analysis revealed a significant positive correlation between Akkermansia and acetic acid, propanoic acid, and NEFA. The Mantel-test network heatmap indicated that Akkermansia not only exhibited a significant positive correlation with acetic acid and propionic acid but also with SCFAs and LA, and its abundance significantly increased in the intestinal tract of doxorubicin-induced HF rats, suggesting that Akkermansia/*A. muciniphila* plays a significant role in the gut microbiota-mediated development of HF. Blautia belongs to the Firmicutes phylum; bacteria of this genus are strictly anaerobic, non-motile, and primarily inhabit the intestinal tracts of mammals, particularly humans (Liu et al., 2021). Blautia species are capable of producing acetate using hydrogen and carbon dioxide during their metabolic processes, a fact confirmed by numerous studies (Rodríguez-García et al., 2024; Wang et al., 2023; Leung et al., 2023; Dai et al., 2021; Kobayashi et al., 2024; Ismaeel et al., 2023; Yu Y. et al., 2019; Niu H. et al., 2024; Wei et al., 2022). Acetate serves not only as a secondary energy source for intestinal epithelial cells but also as an energy source for muscle and brain tissues (Kobayashi et al., 2024; Ismaeel et al., 2023). Moreover, Blautia prevent pathogen colonization through the production of bacteriocins and exhibit anti-inflammatory characteristics and maintenance of glucose homeostasis by upregulating Treg cells and SCFAs production (Yu W. et al., 2019; Niu Y. et al., 2024; Wei et al., 2022). Our results also found a significant positive correlation between Blautia and acetic acid, propanoic acid, NEFA, LA, and a significant negative correlation with ATP, with its abundance significantly increased in the intestinal tract of doxorubicin-induced HF rats. Following treatment, compared to the Model group, the abundance of Akkermansia/*A. muciniphila* showed a trend of recovery in the SFI group, but their abundance further increased in the TMZ group. Interestingly, the abundance of Blautia further increased in the SFI group but showed a trend of recovery in the SFI group. In conclusion, we speculate that SCFAs-producing bacteria may play a significant role in the process of HF by participating in energy metabolism, with Akkermansia and Blautia playing key roles. However, whether the mechanism of action of Shenfu Injection is related to the regulation of key bacteria involved in energy metabolism requires further research and validation.

## Conclusion

This study reveals the interplay between myocardial energy metabolic disorder and gut microbiota dysbiosis during the process of HF. The research findings indicate that the energy supply pattern of cardiomyocytes changes during HF, characterized by a decrease in fatty acid oxidation and an enhancement of anaerobic glycolysis of glucose, leading to insufficient myocardial energy supply. Concurrently, the structure of the gut microbiota is altered, with a reduction in beneficial bacteria, an increase in opportunistic pathogens, and impaired intestinal barrier function. The pharmacological agents Shenfu Injection and trimetazidine used in the experiment both ameliorate myocardial energy metabolism and enhance cardiac function, but Shenfu Injection demonstrates a more significant effect in regulating the gut microbiota and improving gut health. The study also suggests that SCFAs produced by the gut microbiota may be closely associated with myocardial energy metabolism, with Akkermansia and Blautia playing key roles in the progression of HF. These two bacteria can produce SCFAs and participate in energy metabolism, thereby affecting the course of HF. Shenfu Injection treatment may improve myocardial energy metabolism and gut health by modulating the abundance of these key bacteria. This indicates the potential for treating HF by regulating the gut microbiota. Although the study did not conduct fecal microbiota transplantation experiments to directly validate the relationship between “gut microbiota-SCFAs-HF,” its findings provide a new perspective for understanding the role of the gut microbiota in CVDs and theoretical support for therapeutic strategies based on gut microbiota modulation.

## Data Availability

The datasets presented in this study can be found in online repositories. The names of the repository/repositories and accession number(s) can be found in the article/Supplementary material.

## Footnotes

^1^ http://circos.ca/

^2^ http://huttenhower.sph.harvard.edu/LEfSe

^3^ http://bionet.ncpsb.org.cn/batman-tcm/index.php

## Glossary

HF: Heart failure
IBD: Inflammatory bowel disease
CVDs: Cardiovascular diseases
TCM: Traditional Chinese medicine
Normal group: Blank control group
Model group: HF control group
SFI group: Shenfu Injection treatment group
TMZ group: Positive drug control group
SCFAs: Short chain fatty acids
TMAO: Trimethylamine-N-oxide
BAs: Bile acids
LDA: Linear discriminant analysis
PCoA: Principal Co-ordinates analysis
NMDS: Non-metric multidimensional scaling
VIF: Variance inflation factor
OTUs: Operational taxonomic units
LVEF: Left ventricular ejection fraction
LVFS: Left ventricular fraction shortening
NT-proBNP: N-terminal pro-brain natriuretic peptide
PA: Pyruvic acid
LA: Lactic acid
NEFA: Non-esterified fatty acid
MDA: Malondialdehyde
SOD: Superoxide dismutase
ATP: Adenosine triphosphate
*A. muciniphila*: *Akkermansia muciniphila*.
